# Ageing-dependent thiol oxidation reveals early oxidation of proteins with core proteostasis functions

**DOI:** 10.26508/lsa.202302300

**Published:** 2024-02-21

**Authors:** Katarzyna Jonak, Ida Suppanz, Julian Bender, Agnieszka Chacinska, Bettina Warscheid, Ulrike Topf

**Affiliations:** 1 Laboratory of Molecular Basis of Aging and Rejuvenation, Institute of Biochemistry and Biophysics, Polish Academy of Sciences , Warsaw, Poland; 2 CIBSS Centre for Integrative Biological Signalling Research, University of Freiburg, Freiburg, Germany; 3 Biochemistry II, Theodor Boveri-Institute, Biocenter, University of Würzburg , Würzburg, Germany; 4 IMol Polish Academy of Sciences, Warsaw, Poland

## Abstract

Oxidative post-translational modifications of protein thiols are well recognized as a readily occurring alteration of proteins, which can modify their function and thus control cellular processes. The development of techniques enabling the site-specific assessment of protein thiol oxidation on a proteome-wide scale significantly expanded the number of known oxidation-sensitive protein thiols. However, lacking behind are large-scale data on the redox state of proteins during ageing, a physiological process accompanied by increased levels of endogenous oxidants. Here, we present the landscape of protein thiol oxidation in chronologically aged wild-type *Saccharomyces cerevisiae* in a time-dependent manner. Our data determine early-oxidation targets in key biological processes governing the de novo production of proteins, protein folding, and degradation, and indicate a hierarchy of cellular responses affected by a reversible redox modification. Comparison with existing datasets in yeast, nematode, fruit fly, and mouse reveals the evolutionary conservation of these oxidation targets. To facilitate accessibility, we integrated the cross-species comparison into the newly developed OxiAge Database.

## Abstract (precis)

Proteome-wide analysis of thiol oxidation during chronological ageing of yeast reveals evolutionarily conserved proteins as early-oxidation targets with a potential impact on proteostasis regulation.

## Introduction

Proteins are essential for a large variety of functions in an organism. They provide the building blocks of the cell, the basis for cell signalling, metabolic activity, transport of molecules, DNA replication, structure, response to stimuli, and many more activities that are crucial for normal cellular function. Thus, proteins must be protected from damage to confer their cellular function. Intracellular oxidants produced during cellular stress or ageing pose an imminent threat to proteins, lipids, and DNA in the cell (Cooke et al, 2003; Pizzino et al, 2017; Mesika & Reichmann, 2019). High or chronic levels of reactive oxygen species (ROS) are often linked with pathological conditions (Krisko & Radman, 2019). During cellular ageing, increasing endogenous levels of ROS are thought to accelerate the ageing process. In contrast, low levels of ROS were established as messengers in young and proliferating cells. Here, specific oxidation-sensitive amino acid residues become reversibly oxidized to change the function of a given protein temporarily. Thiol groups in the protein cysteine (Cys) residues are known to be reactive and can become readily oxidized (Paulsen & Carroll, 2013). Reversible thiol modifications linked with changes in the function or activity of proteins are commonly referred to as redox switches (Antelmann & Helmann, 2011; Groitl & Jakob, 2014). Many proteome-wide studies in various species mapped oxidation-sensitive cysteine residues, but only for a minority, the biological function of the redox switch is known (McDonagh et al, 2014; Gould et al, 2015; Araki et al, 2016; Topf et al, 2018; van der Reest et al, 2018; Xiao et al, 2020; Meng et al, 2021). Redoxome studies commonly found that cellular proteins are largely present in a reduced state under non-stressed physiological conditions (Go et al, 2011; Brandes et al, 2013; Topf et al, 2018; Meng et al, 2021). Some oxidative thiol modifications are reversed through the action of specific cellular reduction systems that control the proteins’ redox state (Morgan et al, 2013; Winterbourn & Hampton, 2015; Rhee, 2016; Calabrese et al, 2017; Ulrich & Jakob, 2019). Overoxidation of thiols damages the proteins irreversibly and can make them more prone to degradation or aggregation (Paulsen & Carroll, 2013), a phenomenon also observed during biological ageing (David, 2012; Reeg & Grune, 2015; Hartl, 2017; Hipp et al, 2019; Finelli, 2020). Although the cause and consequences of protein damage during ageing have a prevalent place in research, too little attention was paid to the advantages of initially increasing ROS levels during ageing, mediating reversible protein thiol modifications and potential adaptation of stress responses.

Thus far, only a handful of studies have addressed the proteome-wide changes in the protein redox state during eukaryotic ageing. Studies on chronologically aged yeast *Saccharomyces cerevisiae*, strain DBY746 (Brandes et al, 2013), and aged nematode *Caenorhabditis elegans* (Knoefler et al, 2012) revealed a general trend of increased proteome oxidation during ageing. More recent work performed in mice (Xiao et al, 2020) and fruit flies (Menger et al, 2015) argues that the selectivity of the oxidation targets but not the global changes in proteome oxidation might be a cellular strategy to regulate the response to ageing at the later stages of life. The study of Xiao et al (2020) contributes a comprehensive database of more than 9,400 reversibly oxidized proteins in young and old mice, aged 4 and 20 mo, respectively. However, because of the lack of data for the intermediate stages of ageing, it is difficult to address the redox changes in the proteome during the early phase of adult life. Data on thiol oxidation at the time of early ageing might help to resolve the conundrum of the role of reversible oxidation as one of the first signals for the cell to fight the ageing process. Although the previous studies in *S. cerevisiae* (Brandes et al, 2013), *C. elegans* (Knoefler et al, 2012), and *Drosophila melanogaster* (Menger et al, 2015) identified early ageing-dependent changes in protein oxidation, the small number of quantified thiol-containing peptides (∼300 for yeast and fruit fly, ∼150 for worm throughout the entire time course) does not allow visualization of the full temporal redox landscape in an ageing eukaryotic system.

In this study, we provide a large dataset on the *S. cerevisiae* thiol protein oxidation landscape during chronological ageing. It is assumed that restriction of cell proliferation is one of the main causes of age-related accumulation of macromolecular defects, leading to a deterioration of tissues and organ failures (Longo et al, 2012). Therefore, chronological ageing in *S. cerevisiae* is a model system of non-dividing cells, which is commonly used to better understand the mechanisms of human ageing (MacLean et al, 2001). We found that key processes of the protein homeostasis network are targeted for thiol oxidation during the early stages of ageing. We refer to these phenomena as “early oxidation.” Proteins involved in translation, de novo folding, and the ubiquitin–proteasome system (UPS) appeared to be rapidly oxidized before bulk oxidation of the proteome accompanied by cell death occurs. A cross-species comparison of the aged redoxomes showed that similar age-dependent oxidation targets can be found in mice. We integrate our yeast data and the published work in other species and provide the results in the form of a database named “OxiAge” openly accessible to the research community (http://oxiage.ibb.waw.pl). The database enables in-depth studies on the function of evolutionarily conserved cysteine residue oxidation during organismal ageing.

## Results

### Analysis of the redox state of yeast proteome during chronological ageing

**Figure 1.**
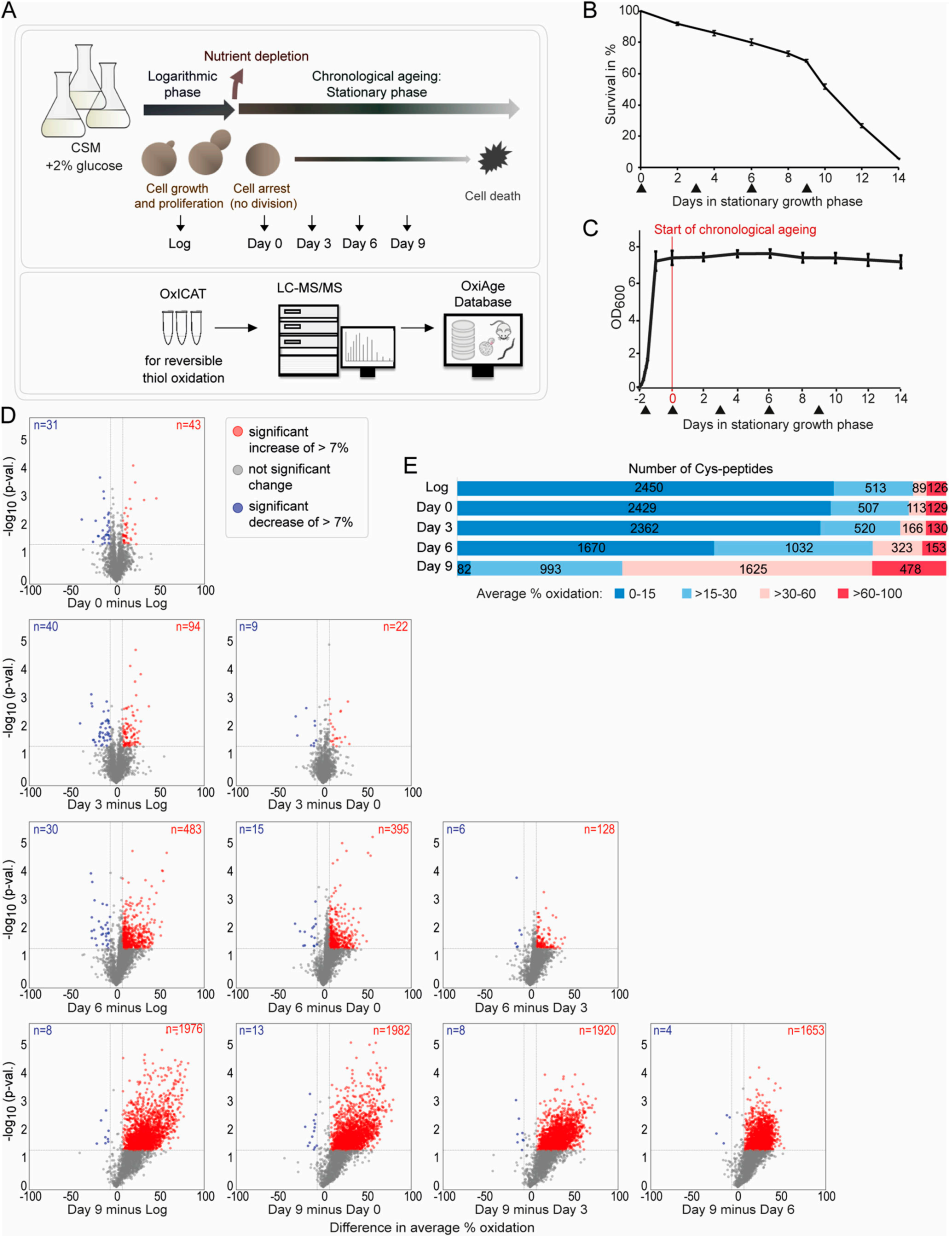
Redox state of yeast proteome changes gradually during progression through chronological ageing. **(A)** Simplified scheme of the experimental design. WT yeast *S. cerevisiae* cells, strain YPH499, were grown in a complete supplement mixture medium with 2% glucose. Cells were collected during the logarithmic growth phase (Log) and four consecutive time points upon entry into the stationary phase (day 0), which marks the start of yeast chronological ageing. Collected samples were subjected to chemical labelling of reversibly oxidized thiols using the OxICAT technique followed by tryptic digestion for LC-MS/MS analysis. Identified peptides were quantified, and the proportion of reversibly oxidized cysteine residues (% oxidation) was calculated. The resulting dataset was compared with data on age-dependent oxidation in other species and integrated into a web application OxiAge Database. **(B)** Lifespan curve of chronologically aged yeast. Samples were collected on indicated days after entry into the stationary growth phase. Cell viability was determined with propidium iodide (PI) staining, and the percentage of dead (stained) cells was given by FACS. At least two biological replicates per time point were analysed. Data are presented as the mean ± SD. Arrowheads below the graph indicate the harvesting time points for OxICAT analysis. **(C)** Growth curve of the YPH499 strain. Time points were taken from the start of the time course at 0 h (day −2, OD600 = 0.1), and at the logarithmic growth at 6, 8, 10, 12, and 24 h (day −1), then during the chronological ageing at 48 h (day 0) and every 48 h until day 14 of the chronological ageing. Six biological replicates were measured. Data are presented as the mean ± SD. Arrowheads below the graph indicate the harvesting time points for OxICAT analysis. *OD*, optical density. **(D)** Pairwise comparison of in vivo cysteine oxidation during the logarithmic phase (Log) and days 0–9 of chronological ageing from the “yeast OxiAge” dataset (with imputed values). Difference in average % oxidation between time points from at least two biological replicates is shown on the *x*-axis; −log_10_ *P*-value (two-tailed Welch’s *t* test) is shown on the *y*-axis. Grey horizontal line, *P*-value = 0.05; grey dots, Cys-peptides of a non-significant difference and/or difference of less than 7%; blue dots, Cys-peptides of a significant decrease of more than 7%; red dots, Cys-peptides of a significant increase of more than 7%. *n*, number of Cys-peptides. **(E)** Distribution of in vivo oxidation status of 3,178 unique Cys-peptides from the “yeast OxiAge” dataset (with non-imputed values). Peptides from each time point were classified into four oxidation groups based on the average % oxidation. Unique peptides per group were counted for each time point. *Log*, logarithmic phase.

**Figure S1.**
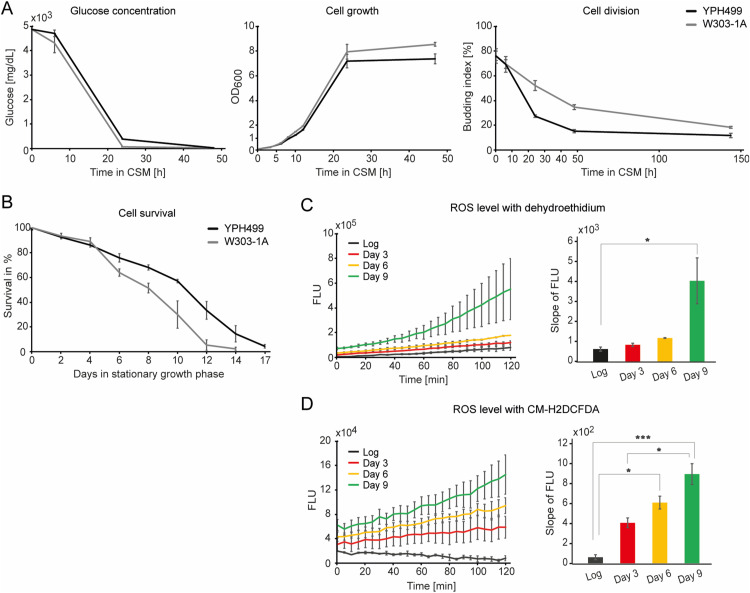
(Related to Fig 1)—Determination of the yeast cell entry into the stationary growth phase. **(A)** Determination of the entry into the stationary phase by measurement of the glucose level (left), growth rate (middle), and budding index (right) in YPH499 and W303-1A yeast strains. Six and four biological replicates were used for YPH499 and W303-1A, respectively. Cell growth of YPH499 during the logarithmic growth phase (middle panel) is part of the data presented in Fig 1C. For glucose measurement, two technical replicates per each biological replicate were taken. The budding index was quantified as % of budding cells for *n* = 200. Data are presented as the mean ± SD. *CSM*, complete supplement mixture medium; *OD*, optical density. **(B)** Lifespan curve of chronologically aged yeast YPH499 and W303-1A. Samples were collected on indicated days after entry into the stationary growth phase. Cell viability was determined with propidium iodide (PI) staining, and the percentage of dead (stained) cells was given by FACS. Six and four biological replicates were analysed for YPH499 and W303-1A, respectively. Data are presented as the mean ± SD. **(C)** Levels of reactive oxygen species in YPH499 measured with dihydroethidium in 5-min time intervals over a time course of 2 h (left) and slope values of the corresponding line graphs (right). Data are presented as the mean ± SD for *n* = 3. *P-value < 0.05 (ANOVA, Tukey’s test). *FLU*, fluorescent units; *Log*, logarithmic phase. **(D)** Levels of reactive oxygen species in YPH499 measured with CM-H2DCFDA in 5-min time intervals over a time course of 2 h (left) and slope values of the corresponding line graphs (right). Data are presented as the mean ± SD for *n* = 3.*P-value < 0.05, ***P-value < 0.01 (ANOVA, Tukey’s test). *FLU*, fluorescent units; *Log*, logarithmic phase.

To investigate the regulation of cellular response to ageing through reversible protein thiol oxidation, we performed a time-resolved, quantitative redoxome study in chronologically aged yeast *S. cerevisiae* strain YPH499 (Fig 1A). We used the established OxICAT method (Leichert et al, 2008) to determine the oxidation status of redox-sensitive protein thiols via quantitative mass spectrometry (MS). We collected samples at the logarithmic growth phase (Log) and the stationary phase of chronologically aged yeast after the diauxic shift (Fig 1A–C). The diauxic shift denotes the transition from fermentation to respiration, using ethanol as a carbon source to drive cell division. As the carbon source becomes depleted, cells shift from the diauxic shift to the G1-arrested stationary phase (MacLean et al, 2001; Herman, 2002; Fabrizio & Longo, 2003). Log samples were collected 6 h after start of yeast growth from OD_600_ = 0.1, at OD_600_ ∼ 0.5 (Fig 1C). YPH499 cells reached OD_600_ > 1 after 10 h and OD_600_ > 7 after 24 h (Fig S1A, middle). We determined the entry into the stationary phase and the commencement of chronological ageing as the state marked by complete glucose depletion from the medium, inhibited cell proliferation (budding index < 15%), and stabilized growth rate at OD_600_ ∼ 7 (Fig S1A). We marked this time point as “day 0” of chronological ageing (Fig 1A–C). Cell survival during chronological ageing was monitored until day 14 for the yeast cultures used in the MS analysis (Fig 1B). The mean lifespan was determined to be >10 d and validated through an independent experiment (Fig S1B). The chosen time frame for sample collection for the MS analysis was based on the survival rate during chronological ageing of yeast strain YPH499. We identified YPH499 as a longer lived yeast strain when compared to other commonly used laboratory strains such as W303-1A (Fig S1B) and previously reported strains DBY476 (Brandes et al, 2013), BY4741 (Czachor et al, 2020), and other (Fabrizio & Longo, 2003; Herker et al, 2004; Qin & Lu, 2006; Li & Snyder, 2016).

**Figure S2.**
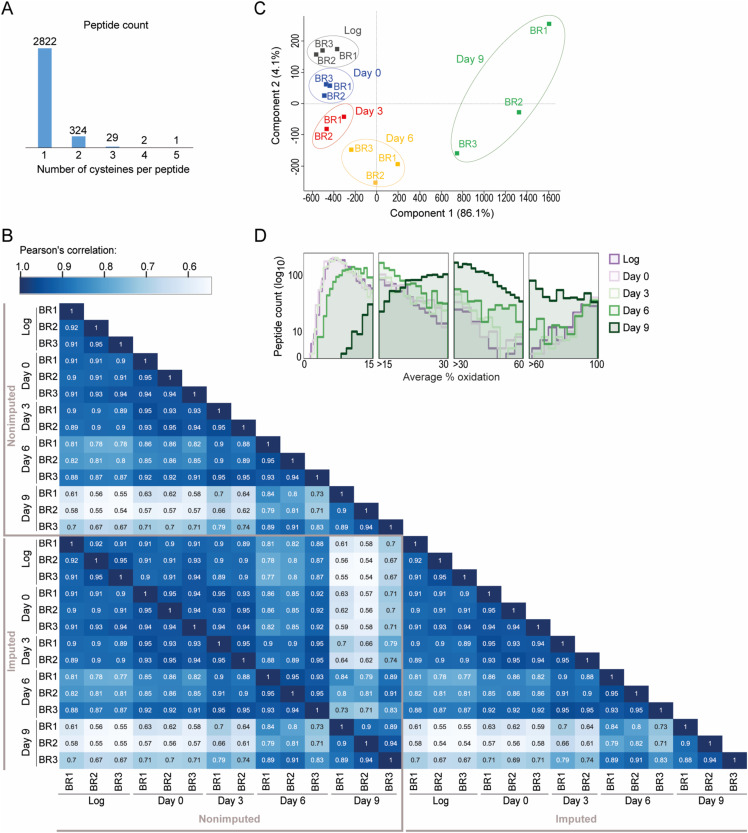
(Related to Fig 1)—Analysis of the “yeast OxiAge” dataset. **(A)** Number of cysteine residues found within a quantified Cys-peptide. Most of the peptides (>2,800) contain only one redox-sensitive cysteine detected and quantified. **(B)** Heatmap of Pearson’s correlation between different biological replicates and time points for not imputed (“Non-imputed”) and imputed (“Imputed”) values. *BR*, biological replicate; *Log*, logarithmic phase. **(C)** Principal component analysis plot showing variation between individual biological replicates and time points for the “yeast OxiAge” dataset with imputed values. *BR*, biological replicate; *Log*, logarithmic phase. **(D)** Histogram presenting log_10_ of distribution of the % oxidation in each group from Fig 1E for each time point. *Log*, logarithmic phase.

The experimental design enabled the monitoring of the reversible oxidation of protein thiols at specific phases of ageing: in young proliferating cells (Log, logarithmic phase), at the entry into the stationary phase (day 0), during early ageing (day 3), mid-point of ageing (day 6), and late stage of ageing (day 9). We detected a total of 9,213 unique cysteine-containing peptides (Cys-peptides) in 3,139 proteins (of 5,478 cysteine-containing proteins described in the yeast proteome, UniProtKB; see the Materials and Methods section) in three biological replicates (each measured in two technical replicates) per time point (Table S1). Because of the low accuracy of the third biological replicate of day 3 and an unproportionally high number of not quantified peptides in comparison with other replicates, we removed this replicate from further downstream analysis. Thus, time point “day 3” was analysed in only two biological replicates. To increase the robustness of our dataset, we retained only Cys-peptides quantified in at least two biological replicates of each time point. The final filtered dataset contains a total of 3,178 quantified thiol-containing peptides, matching 3,564 cysteine sites on 1,772 proteins (Table S2). Among the quantified Cys-peptides, 2,822 peptides contain a single cysteine residue, 324 peptides two residues, and 32 peptides at least three residues (Fig S2A). In the following, we will refer to the filtered dataset as the “yeast OxiAge” dataset (Table S2). The quantified redox changes were highly reproducible between biological replicates of each time point with average Pearson’s correlation coefficients of 0.93 for the dataset without imputed values and the dataset containing in addition imputed missing values (Fig S2B). Principal component analysis (PCA) showed that biological replicates were separated between the different time points (Fig S2C).

Table S1 Source file of mass spectrometry data of all detected Cys-peptides containing redox-sensitive thiols at different time points of chronological ageing in WT yeast *S. cerevisiae*, strain YPH499.

Table S2 Filtered mass spectrometry data of quantified Cys-peptides containing redox-sensitive thiols at different time points of chronological ageing in WT yeast *S. cerevisiae*, strain YPH499. We refer to this data as “yeast OxiAge.”

To identify peptides with a significant change in oxidation, we compared the levels of thiol oxidation of quantified peptides between each time point of the experiment (Fig 1D). We refer to the proportion of reversibly oxidized cysteine residues as “% oxidation” or “oxidation level.” Minor changes in the oxidation level occurred until day 3 of ageing, with a comparable number of peptides exhibiting an increased or decreased % oxidation. On day 3, 94 Cys-peptides exhibited a significant increase in oxidation of >+7% compared with the Log (Fig 1D, in red), whereas 40 Cys-peptides showed a significant decrease of >−7% oxidation (Fig 1D, in blue). At day 6 of ageing, a visible shift towards higher oxidation of cysteine thiols occurred. At least 16 times more Cys-peptides showed a significant increase rather than a decrease of >7% in oxidation between day 6 and earlier days of ageing. This shift was even more pronounced on day 9, concomitant with a rapid increase in cell lethality (Figs 1B and S1B). The observed gradual increase in reversible proteome oxidation correlates with an increase in the cellular levels of ROS (Fig S1C and D).

The manual classification into four groups of % oxidation, 0–15%, >15–30%, >30–60%, and >60–100%, revealed that the basal level of oxidation in the logarithmic phase is below 15% for 2,450 peptides (Figs 1E and S2D). This confirms previous analyses performed in proliferating yeast, demonstrating that in unstressed environmental conditions, cysteine residues are largely present in a reduced state (Topf et al, 2018). 126 Cys-peptides had an oxidation of more than 60% in the logarithmic growth phase, and this number is comparable with days 0 and 3, with a mild increase at day 6 (Fig 1E). Most changes occurred for peptides with an oxidation state above 15% and in the >30–60% range. Notably, the distribution of average % oxidation of Cys-peptides quantified at later stages of ageing (days 6 and 9) exhibits a distinct pattern in all four groups, trending towards higher oxidation states when compared to the Log and days 0 and 3 (Fig S2D). Peptides quantified for day 6 demonstrate a notable shift towards the upper range in the 0–15%, >15–30%, and >30–60% groups, in contrast to earlier days.

Thiol-containing peptides that are oxidized at a low level in young proliferating cells exhibit a visible shift to a higher redox state during the early days of ageing. Between the young cells and aged cells from days 3 and 6, the shift was observed mostly from low oxidation (below 15%) to medium oxidation (>15–60%). Thus, changes in oxidation during ageing were mostly gradual. Only at the late phase of ageing (day 9), the number of highly oxidized peptides increased dramatically, which is likely accompanied by oxidative damage.

### Classification of redox changes in the ageing proteome

**Figure 2.**
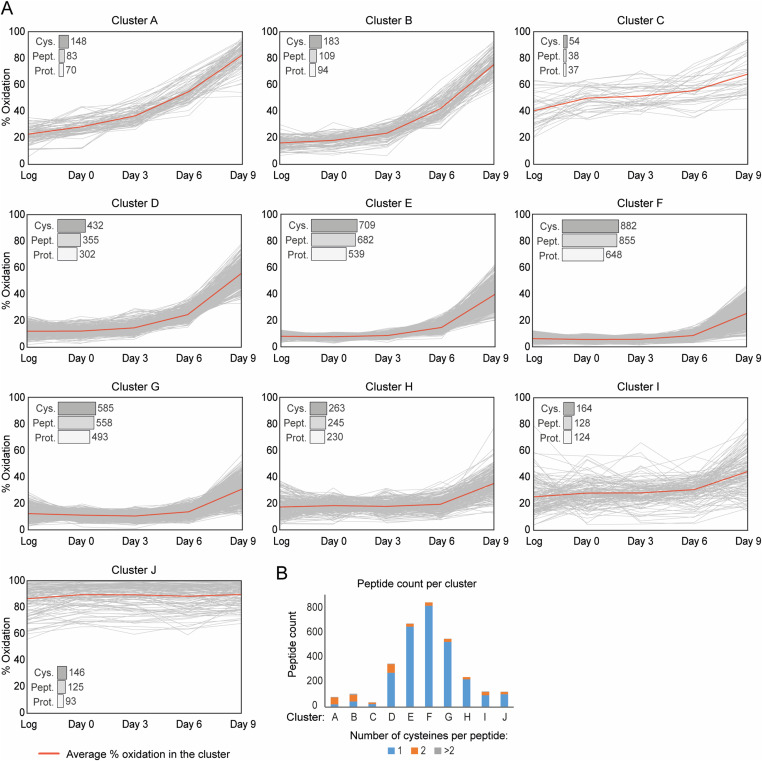
Classification of temporal changes in proteome oxidation based on peptide-specific patterns. **(A)** Graphs indicating the average % oxidation per Cys-peptide over time. Cys-peptides from the “yeast OxiAge” dataset were clustered into 10 groups, named A–J, of distinct oxidation patterns. Peptides within clusters A–D exhibit the fastest increase in % oxidation. Clusters E–H contain peptides with distinct elevation in % oxidation during the late phase of ageing on day 9. Peptides from cluster J have stable and high oxidation levels. Cluster I contains peptides with various redox patterns that could not be automatically classified into other groups. Grey traces, average % oxidation for each Cys-peptide from at least two biological replicates at the specified time point; red trace, average % oxidation within the cluster. *Log*, logarithmic phase; *Cys.*, the number of unique cysteine residues within the cluster; *Pept.*, the number of unique Cys-peptides within the cluster; *Prot.*, the number of unique proteins within each cluster. Note that each Cys-peptide is specific to one cluster. A unique cysteine residue can be assigned to more than one cluster if the residue is present in more than one Cys-peptide. Each protein can be assigned to more than one cluster if the protein has more than one Cys-peptide quantified and grouped into different clusters. **(B)** Number of cysteine residues found within a quantified Cys-peptide per each cluster A–J.

**Figure S3.**
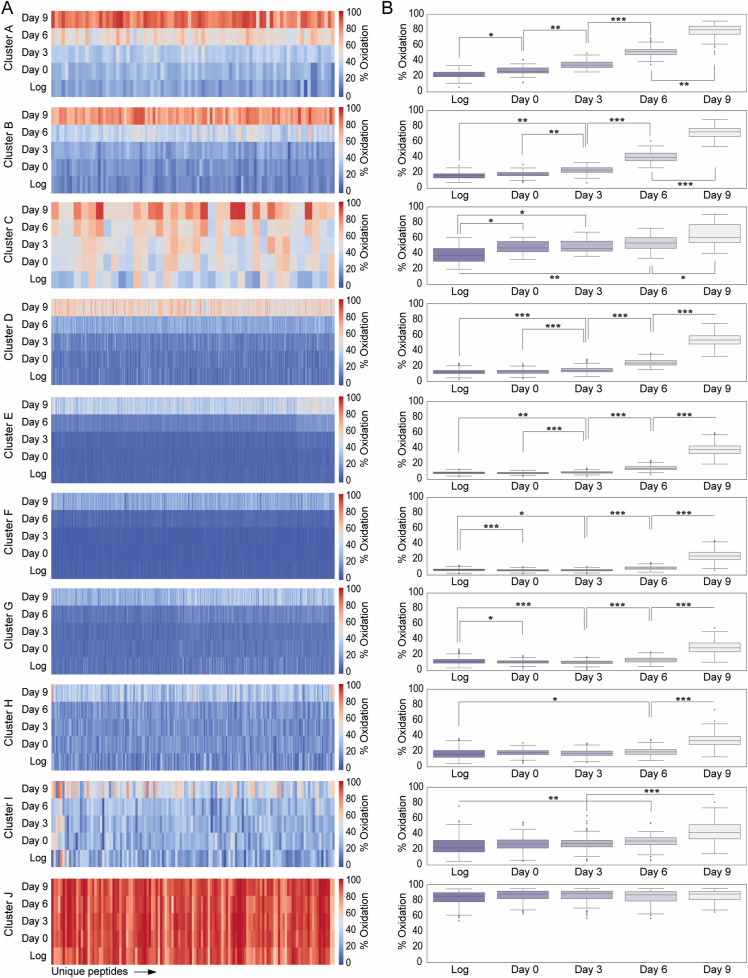
(Related to Fig 3)—Distinct patterns of sequential Cys-peptide oxidation for individual clusters. **(A)** Heatmap of average % oxidation per Cys-peptide on each day for each cluster, A–J. *Log*, logarithmic phase. **(B)** Boxplots of average % oxidation per Cys-peptide on each day for each cluster, A–J. Statistical significance of the difference in % oxidation between clusters was assessed using the Kruskal–Wallis test with Dunn’s post hoc method with the Bonferroni correction: *P-value < 0.005, **P-value < 0.00005, ***P-value < 0.0000005. *Log*, logarithmic phase.

To distinguish between different trends in thiol oxidation and identify proteins that are most sensitive to the age-dependent increase in oxidative stress, we performed a clustering analysis of our time-resolved thiol oxidation data. The clustering resulted in 10 distinguished groups (cluster A–J) of thiol-containing peptides based on the average % oxidation at the different time points and the oxidation pattern (Figs 2A and S3A and B, Table S3). Most of the time points per cluster contain data with an interquartile range (IQR) < 7, indicating small variability between grouped peptides per cluster (Fig S3B).

Table S3 Information on clusters A–J to which individual Cys-peptides from the “yeast OxiAge” dataset were assigned. Along with cluster names, the file provides information on amino acid sequences surrounding oxidized cysteine residue and protein localization.

Clusters A, B, C, and D contain peptides with cysteine residues showing a considerable increase in % oxidation at days 3 and 6 when compared to the Log. These clusters contain in total 585 Cys-peptides of 458 unique proteins. More than 50% of Cys-peptides from clusters A and B contain at least two cysteine residues (Fig 2B). The earliest increase in % oxidation is observed in clusters A, B, and C. Cluster A contains 83 peptides, corresponding to 148 unique cysteine residues and 70 unique proteins (Fig 2A). These peptides were significantly more oxidized on day 3 than during the Log (corr. *P*-val. < 0.00005, Dunn’s test), with an increase from ∼20% to ∼35% and a further increase on day 6 to average oxidation of ∼55% (corr. *P*-val. < 0.0000005, Dunn’s test) (Fig S3B). For cluster B, Cys-peptides showed a delay in increase of % oxidation compared with cluster A. Globally, a significant increase was observed already on the 3rd d of ageing (corr. *P*-val. < 0.00005, Dunn’s test). Intriguingly, we detected the earliest increase in % oxidation for peptides in cluster C. This cluster contains Cys-peptides that, on average, were oxidized >40% during the Log. The cluster is characterized by a strong variation in % oxidation between Cys-peptides (Fig S3A).

Most thiol-containing peptides are grouped into clusters E–H, with no or little changes in % oxidation during early time points (Fig S3A and B) and a small number of peptides containing more than one cysteine residue (Fig 2B). On average, the oxidation of Cys-peptides was <20% during the early and mid-point of ageing. Cys-peptides from cluster G exhibited a decrease in oxidation when entering the stationary phase of day 0, and then a sequential increase in the later days. The maximum thiol oxidation in clusters E–H on day 9 was on average <50%. Thus, protein thiols within these clusters can be considered less sensitive to oxidation-dependent modifications during ageing.

Several peptides grouped into a non-homogeneous cluster I, containing 128 Cys-peptides in 124 proteins, 55 of which were unique to this cluster (Fig 2A). In general, cluster I consists of Cys-peptides that could not be grouped into any other cluster and did not exhibit any consistent trend. Within this cluster, there are peptides of proteins that may be considered reversibly oxidized during early ageing and irreversibly oxidized or degraded in the older yeast culture.

Custer J contains 125 Cys-peptides corresponding to 93 proteins that did not change their redox status and remained highly oxidized (>85%) over the time course (Fig 2A). 80 proteins contained at least one Cys-peptide assigned exclusively to cluster J, whereas 13 proteins contained peptides allocated in addition in clusters C–I. Intriguingly, no protein with Cys-peptides assigned to clusters A or B was present in cluster J.

In summary, whereas most protein thiols within the “yeast OxiAge” dataset were oxidized late during ageing, we identified proteins that contained cysteine residues sensitive to changes in oxidation during the early phase of ageing. We hypothesize that these proteins are likely to influence cellular response and organismal fate during biological ageing.

### Peptides with cysteine residues oxidized during early ageing exhibit a common amino acid signature

**Figure 3.**
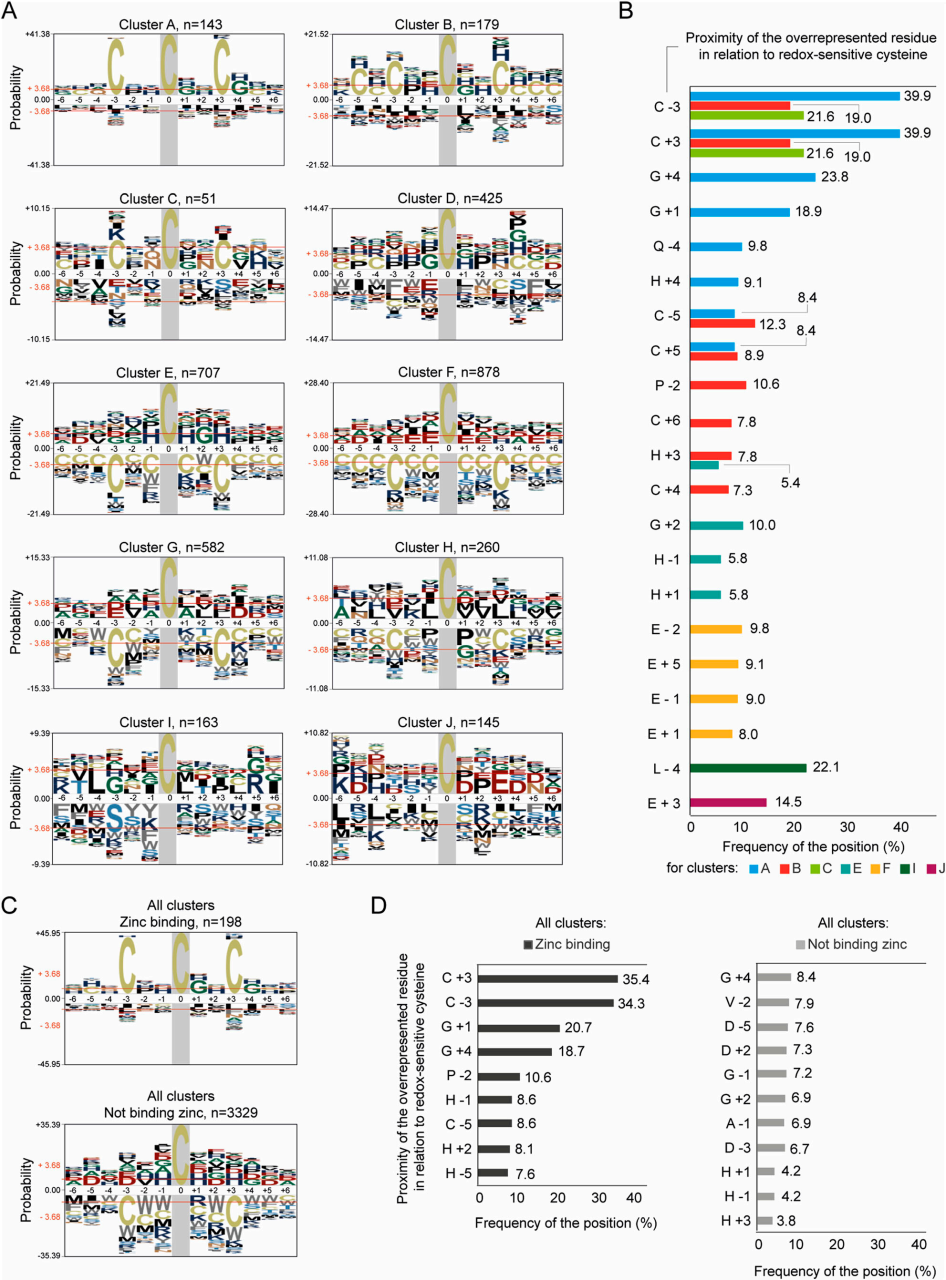
Motif analysis of oxidized cysteine residues demonstrates a conserved sequence in individual clusters. **(A)** Consensus motifs for reversibly oxidized cysteine residues for Cys-peptides within clusters A–J. Clusters A, B, and C show a significant overrepresentation of cysteine residues at either position −3 or +3, whereas clusters E–H show a significant selection against cysteines at the same positions. Graphs present the log-odds binomial probabilities. The sum of the log-odds probabilities for the largest column is used as the maximum value for the *y*-axis. Residues are scaled proportionally to their log-odds binomial probabilities under the chosen background with the biggest residue indicating the strongest statistical significance. Amino acid residues are coloured according to their physicochemical properties. Red horizontal line at ±3.68, *P*-value = 0.05 (Bonferroni-corrected). Probabilities above +3.68, overrepresented statistically significant residues; probabilities below −3.68, underrepresented statistically significant residues. Grey area, fixed position of the oxidized cysteine residue. Column numbers, positions from the centred oxidized cysteine at position 0. *n*, number of unique motifs analysed in individual clusters for each oxidized cysteine site. **(B)** Frequency in % of the significantly overrepresented amino acid residues at specified positions in relation to the oxidized cysteine within the individual clusters. Only clusters A, B, C, E, F, I, and J contain significantly enriched motifs. Columns indicate the residue (*C*, cysteine; *G*, glycine; *Q*, glutamine; *H*, histidine; *P*, proline; *E*, glutamic acid; *L*, leucine) and its position relative to the oxidized cysteine. **(C)** Consensus motifs for zinc-binding and non-binding redox-sensitive cysteines within all clusters. Significant overrepresentation of the CXXC motif is observed for zinc-binding residues. **(D)** Frequency in % of the significantly overrepresented amino acid residues at specified positions in relation to the oxidized zinc-binding or non-binding cysteine. Columns indicate the residue (*C*, cysteine; *G*, glycine; *H*, histidine; *P*, proline; *D*, aspartic acid; *A*, alanine; *V*, valine) and its position relative to the oxidized cysteine.

We asked whether the early-oxidized Cys-peptides exhibit any significant similarities that might affect their sensitivity to oxidation. We analysed the local amino acid environment of the early-oxidized cysteine residues (Table S3, “Motifs per cluster”). We found that a common feature of the peptides assigned to clusters A, B, and C was the presence of additional cysteine residues surrounding the early-oxidized one (Fig 3A). Within Cys-peptides that showed an increase in oxidation on days 0 and 3, an additional cysteine in close proximity to the oxidation-sensitive cysteine residue was overrepresented in the peptide sequence. A second cysteine was present either at position −3 or +3 from the oxidized cysteine subjected to motif analysis. We found that 44 of 83 Cys-peptides in cluster A contained two cysteine residues that both formed the CXXC motif. Subjecting both cysteines to the motif analysis led to the generation of a symmetric signature, where the same cysteine might be present at position 0, −3, or +3, depending on the analysed residue (position 0). In the case of 17 Cys-peptides, a single cysteine from the CXXC motif was found to be early-oxidized within cluster A. Among them, two proteins contained both cysteine residues oxidized and found within two different Cys-peptides: Rpa135 (Cys1104 and Cys1107) and Gfa1 (Cys183 and Cys186). Interestingly, two proteins, the stress response protein Nst1 and the aminopeptidase Fra1, showed a CXXCXXC motif with all three cysteine residues present within the same early-oxidized Cys-peptide. The CXXC motif was also found for Cys-peptides in clusters B and C, but a cysteine residue either at position −3 or +3 occurred with a lower frequency than in cluster A (Fig 3B). The CXXC motif has been previously identified for cysteine residues responding to the treatment with H_2_O_2_ with a significant increase in oxidation (Topf et al, 2018) and during ageing (Brandes et al, 2013). Topf et al (2018) noted that this motif was also associated with the binding of zinc ions. Our analysis confirmed the significant overrepresentation of the CXXC motif in zinc-binding proteins, similar to early-oxidized proteins from clusters A–C (Fig 3C and D).

Early-oxidized Cys-peptides in cluster A had a tendency for the enrichment of a non-polar amino acid glycine (Gly/G) at positions +1 and +4 with a frequency of ∼20% (Fig 3B). We observed a smaller frequency for a positively charged histidine at position +4 (His/H; frequency ∼9%) and polar glutamine at position −4 (Glu/Q; frequency ∼10%). Cysteine residues in cluster B showed a tendency for more complex motifs composed of several additional Cys/C in proximity: 3, 4, 5, or 6 positions away from the oxidized cysteine (Fig 3A; cluster B). Peptides grouped in cluster D, which showed mid-point oxidation during ageing, did not exhibit a specific motif (Fig 3A; cluster D). Interestingly, Cys-peptides within the clusters E–H, which were oxidized during late ageing, showed a significant underrepresentation of motifs with multiple cysteine residues in proximity. Although overrepresentation of any particular amino acid sequence was less common in these clusters, cluster F contained a significant enrichment for glutamic acid in proximity to late-oxidized cysteine residues (Glu/E; position ±1; frequency ∼20%) (Fig 3B). Here, we found an additional acidic residue at positions −2 and −5 (frequency < 10%). Interestingly, we observed a trend of selection for an acidic amino acid in the “yeast OxiAge” dataset for peptides of high redox modification independent of cellular age (cluster J), with enrichment for Glu/E, and close to the significant overrepresentation of aspartic acid (Asp/D) (Fig 3A and B; cluster J).

It is worth noting that identifying unique cysteine motifs of peptides that exhibit higher sensitivity to oxidative modification during early phases of ageing might serve as a starting point for finding potential cysteine residues that rapidly respond to oxidative stress related to chronological ageing.

### Peptide classification reveals distinct functional groups of early-oxidized proteins

**Figure 4.**
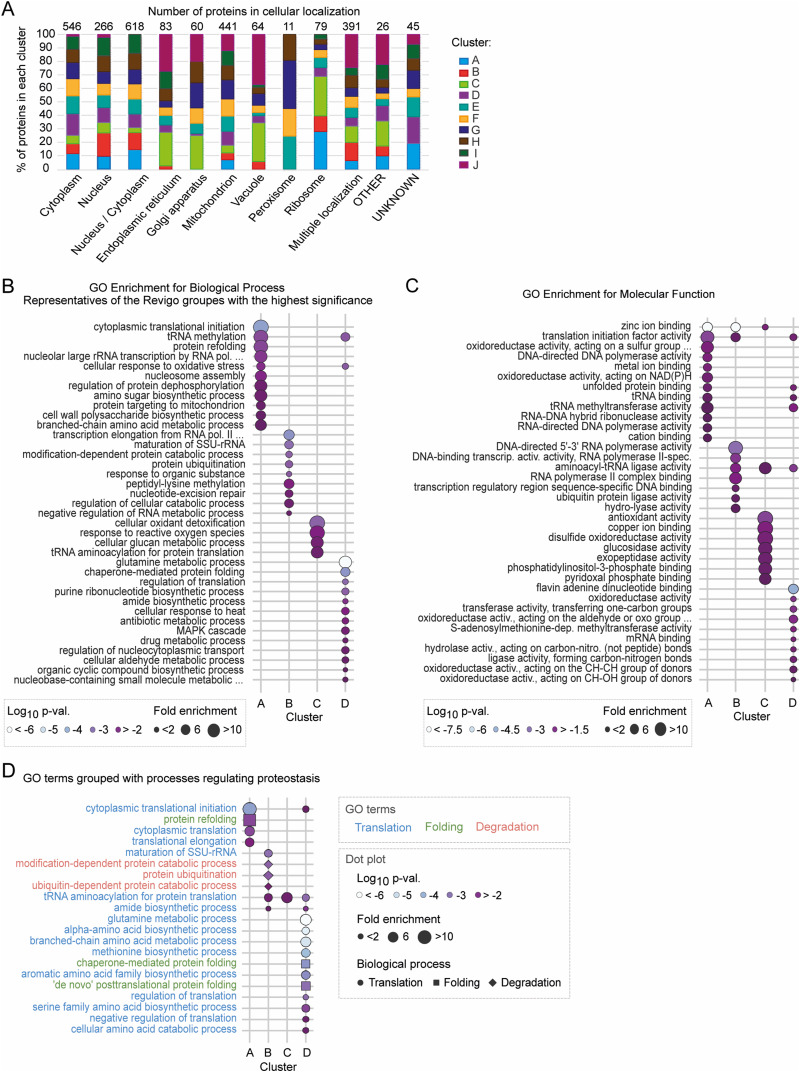
Distinct functional groups of proteins oxidized at different stages of ageing. **(A)** Distribution of proteins with at least one Cys-peptide assigned to individual clusters according to subcellular localization. The frequency of proteins within a particular cluster is presented for each localization. Gene Ontology (GO) terms for cellular components were retrieved from UniProtKB (see the Materials and Methods section). The total number of proteins per localization is indicated above the bar plots. **(B)** GO term enrichment analysis of biological process (BP) for individual clusters A–D. For simplification, GO BP terms were grouped based on semantic similarity (Revigo tool). Depicted GO BP are representative of the groups with the highest statistical significance (Fisher’s exact test, Benjamini–Hochberg correction). 15 best-scored terms are shown. Dot size, fold enrichment; dot colour, log_10_ uncorrected *P*-value (Fisher’s *t* test). **(C)** GO term enrichment analysis of molecular function (MF) for individual clusters A–D. A maximum of 15 best-scored terms based on the highest statistical significance (Fisher’s exact test, Benjamini–Hochberg correction) are shown. **(D)** GO BP terms for clusters A–D representing processes involved in proteostasis regulation or grouped by semantic similarity with such terms (see the Materials and Methods section). Colours of GO term indicate the proteostasis process: pastel blue, cytoplasmic translation; pastel green, protein folding; pastel red, protein degradation. Marker size, fold enrichment; marker colour, log_10_ uncorrected *P*-value (Fisher’s exact test); marker shape, biological process of proteostasis: circle, cytoplasmic translation; square, protein folding; diamond, protein degradation.

**Figure S4.**
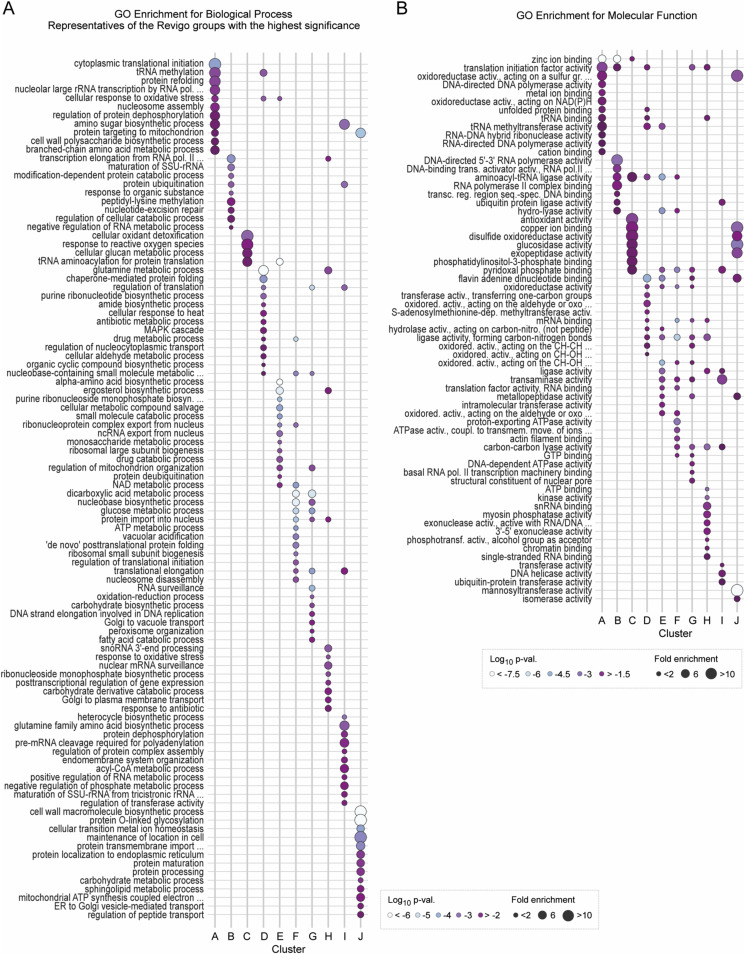
(Related to Fig 4)—GO enrichment analysis of biological process and molecular function for individual clusters. **(A)** GO term enrichment analysis of biological process (BP) for individual clusters A–J. GO BP terms were grouped by semantic similarity (Revigo tool). GO BP terms shown are representative of the Revigo groups with the highest statistical significance (Fisher’s exact test, Benjamini–Hochberg correction). A maximum of 15 best-scored terms are shown. Dot size, fold enrichment; dot colour, log_10_ uncorrected *P*-value. **(B)** GO term enrichment analysis of molecular function (MF) for individual clusters A–J. A maximum of 15 best-scored terms based on the highest statistical significance (Fisher’s exact test, Benjamini–Hochberg correction) are shown. Dot size, fold enrichment; dot colour, log_10_ uncorrected *P*-value.

To identify the function of the proteins within the distinct clusters, we analysed their annotated subcellular localization (Fig 4A, Table S3, “Peptides and Localization”) and performed a Gene Ontology (GO) overrepresentation analysis of the terms “biological process” (BP) and “molecular function” (MF) (Figs 4B and C and S4A and B, Table S4). We did not observe any correspondence between peptide clusters and subcellular compartments. An exception was cluster J, which is enriched in proteins localized in the endoplasmic reticulum (ER), Golgi apparatus, mitochondrion, and vacuole (Fig 4A). Peptides within clusters C and J are characterized by exceptionally high oxidation states already in proliferating cells. Indeed, the cellular organelles mentioned above have a higher redox potential compared with, for example, the cytoplasm (Go & Jones, 2008). A large part of early-oxidized Cys-peptides belonging to cluster A correspond to proteins allocated to ribosomes. This is consistent with GO enrichment analysis showing a significant enrichment in cytoplasmic translation processes (Fig 4B) and translation activities (Fig 4C) within this cluster. An example of an early-oxidized protein regulating cytoplasmic translation is Tif35 (human eIF3g), a subunit of translation initiation factor eIF3. One quantified peptide of Tif35 contains two cysteine residues at positions 112 and 121, which form a disulphide bond. A change in % oxidation of this Cys-peptide was fast with an increase from 25.8% ± 8.7% on day 0 to 42.4% ± 5.4% on day 3. We also detected tRNA synthetases within cluster A. Among them was Mes1 (human MetRS/MARS1) with two cysteine sites oxidized during the early stages of ageing. Cys353 had a basal oxidation level of 22% ± 4.5% during the Log that elevated to 37.8% ± 5% on day 3. The consecutive time points followed this trend with an increase to 62% ± 11.7% and 92.9% ± 4.8% on days 6 and 9, respectively. As suggested by structure prediction (AF-P00958-F1, AlphaFold prediction [Jumper et al, 2021]), this residue is localized on the surface of the protein and interacts with three other residues at positions 337, 340, and 350. We detected the second thiol in Cys321 oxidized later than the other cysteine, and thus grouped within cluster D.

Table S4 Gene Ontology (GO) enrichment for biological process (BP) and molecular function (MF) of proteins assigned to clusters A–J in the “yeast OxiAge” dataset.

Furthermore, we found that the biological processes “protein refolding” and “ribosomal RNA (rRNA) transcription by RNA polymerase I” were strongly enriched in cluster A (Fig 4B). We identified six unique cysteine sites of RNA polymerase I subunit Rpa135 (human RPS135/POLR1B). We found Cys1104 and Cys1107 in two different peptides grouped into cluster A, with oxidation of ∼20% in proliferating cells, later increasing to ∼30% on days 0 and 3 and >44% on day 6. Cys1107 of Rpa135 was reported to be essential for recruiting Rpa190, the largest subunit of RNA polymerase I (Naryshkina et al, 2003). Cysteine residues at positions 158, 584, 585, and 734 of Rpa135 were grouped into cluster D with an increase in oxidation on days 3 and 6. Another protein with early-oxidized cysteine residues (positions 40, 43, 68, and 73) is Mig1, involved in glucose repression. Activation of Mig1-repressed genes is critical for maintaining chronological ageing in yeast (Maqani et al, 2018). It is strictly regulated by the phosphorylation of multiple serine residues, but the possibility of its regulation through cysteine residue modification has so far not been described.

Contrary to cluster A, cluster B contains proteins mostly found to regulate protein degradation and mRNA transcription (Fig 4B). An example of a protein involved in the latter process is a subunit of RNA polymerase II, Rpb9 (human RPB9/POLR2I), with a single thiol group on Cys32 oxidized from 16.6% ± 4.1% during the Log to 34% ± 7.1% on day 3. This fast oxidation was followed by a continuous increase to 44.8% ± 3.9% and 71.9% ± 12.6% on consecutive days. Consistently, a lower increase in % oxidation until the mid-point of ageing (<60%) might indicate a possible oxidation-dependent regulation of the protein in older yeast culture.

Cluster C was significantly overrepresented for proteins involved in response to oxidation, glucan metabolism, and tRNA aminoacylation for translation (Fig 4B). A prominent example in cluster C is the glutathione-dependent disulphide oxidoreductase Grx1 (human GLRX and GLRX2), which protects against oxidative damage (Luikenhuis et al, 1998). Cys27 and Cys30 were present in a single peptide with strong oxidation of more than 40% in proliferating cells, and subsequent increase on day 0 to ∼50% with no significant change on day 3. A strong increase to more than 70% was observed on day 6. Both cysteine residues form the redox-active CXXC motif of the protein. When Grx1 reduces a disulphide bond in a substrate protein, it results in the formation of a catalytic disulphide bond between these cysteines. This catalytic disulphide bond can subsequently be reduced by glutathione (Begas et al, 2017). Catalytic disulphides, as well as structural disulphide bonds, are also formed within Ero1, a thiol oxidase essential for oxidative protein folding in the ER. A peptide containing Cys166 of Ero1 grouped within cluster C. It was shown previously to form a disulphide bond with Cys143 important for the structure of Ero1 (Sevier et al, 2007). Another example of cluster C protein is the methionine aminopeptidase Map1, which localizes to cytosolic ribosomes and catalyses the cotranslational removal of N-terminal methionine from nascent polypeptides. This included the peptide containing Cys22 and Cys27. Both cysteine residues are part of a zinc finger motif (together with Cys37 and Cys40). Mutation of Cys22 to serine was shown to decrease Map1 ribosome association in vivo (Vetro & Chang, 2002) but did not result in a decrease in Map1 catalytic function in vitro (Zuo et al, 1995). Thus, it was suggested that the zinc finger is involved in the correct positioning of Map1 on the translationally active ribosomes (Vetro & Chang, 2002). Possibly, oxidation of Cys22 or Cys27 could also affect the integrity of the zinc finger domain.

Within cluster D, we detected significant enrichment of proteins involved not only in glutamine metabolism, but also in chaperone-mediated protein folding and regulation of translation (Fig 4B). Similar to cluster A, proteins linked to translation initiation factor activity, tRNA binding, and unfolded protein binding were present (Fig 4C).

Proteins with peptides identified in clusters E–H represented similar biological processes (Fig S4A and B). For example, clusters E and F were significantly enriched for a global response to oxidative stress, tRNA aminoacylation, amino acid biosynthesis, and ribosomal biogenesis. This includes some ribosomal proteins, such as Rps11B (Cys58 and Cys128) or Rps8B (Cys179; Cys168 in cluster G). We identified a variety of regulators of protein folding, such as chaperonin-containing TCP-1 (CCT) multiprotein complex components and heat shock proteins (HSP). This characteristic was shared between clusters E, F, and G. In addition, mitochondrion organization and metabolic processes were among the significantly enriched biological processes.

Cys-peptides in clusters I and J correspond to proteins involved in a variety of biological processes relevant to cellular metabolism (Fig S4A). The analysis of the cellular compartmentalization of proteins grouped in cluster J revealed that they are preferentially localized in the mitochondrion (16 proteins) and vacuole (13 proteins) (Fig 4A). Accordingly, proteins assigned to cluster J are involved in protein targeting to the mitochondrion, cell wall organization, glycosylation, and general regulation of intercellular transport (Fig S4A). An example of such a protein is the vacuolar aspartyl protease Pep4. We found three cysteine residues at positions 122, 127, and 328, oxidized above 80% over the yeast’s lifespan. All oxidized cysteine sites are localized within the peptidase A1 domain and are involved in disulphide bond formation. Another example is the mitochondrial intermembrane space import and assembly protein 40 (Mia40) (Chacinska et al, 2004). For this protein, Cys317 identified in this study is one of six known cysteine sites involved in the formation of disulphide bonds.

In agreement with the motif analysis, we found significant enrichment for zinc-binding proteins in clusters A, B, and C (Fig 4C). We did not observe this enrichment in any other cluster of Cys-peptides oxidized at later stages of ageing (Fig S4B). The zinc-binding proteins identified within early-oxidation clusters align with previously identified proteins sensitive to oxidative stress (Topf et al, 2018). Notably, clusters C and J containing strongly oxidized peptides already during the proliferation stage were significantly enriched in proteins known for binding copper ions (Fig S4B). These proteins are predominantly localized in mitochondria (Fig 3A) and are recognized for their role in maintaining redox homeostasis (see below). Four copper-binding mitochondrial proteins are identified among those highly redox-sensitive assigned to cluster J. Superoxide dismutase Sod1, a key player in antioxidant defence, converts superoxide radicals to oxygen and hydrogen peroxide (Eleutherio et al, 2021). Fet5, an iron transport multicopper oxidase, participates in copper-dependent iron transport, indicating a connection between copper availability and iron transport processes (Spizzo et al, 1997). Moreover, two other copper-binding proteins, Cox17 and Coa6, are involved in cytochrome c oxidase assembly, suggesting a potential link between copper binding and mitochondrial function (Glerum et al, 1996; Ghosh et al, 2014). Additional two copper-binding proteins, Cup1-2 (a copper metallothionein) and Ccs1 (a copper chaperone for Sod1), have cysteine residues oxidized at the entry into the stationary phase (“day 0”), grouped within cluster C. Both proteins are involved in sensing ROS levels and directing the activation of Sod1. Cup1 exhibits antioxidant activity, contributing to the detoxification of oxygen radicals, particularly in the absence of Sod1 (Tamai et al, 1993). Notably, only Ccs1 is annotated to bind copper to its cysteine residues. Oxidation of the cysteine sites of Ccs1 may impact the proper delivery of copper ions to Sod1, influencing its antioxidant defence function (Culotta et al, 1997; Lamb et al, 2000).

The functional analysis of proteins carrying redox-sensitive thiols revealed a specific pattern. It appears that proteins involved in key cellular processes of translation, folding, and degradation contain cysteine residues that are preferably oxidized during the early stages of chronological ageing.

### Oxidation of proteins regulating proteostasis as an early response to ageing

**Figure 5.**
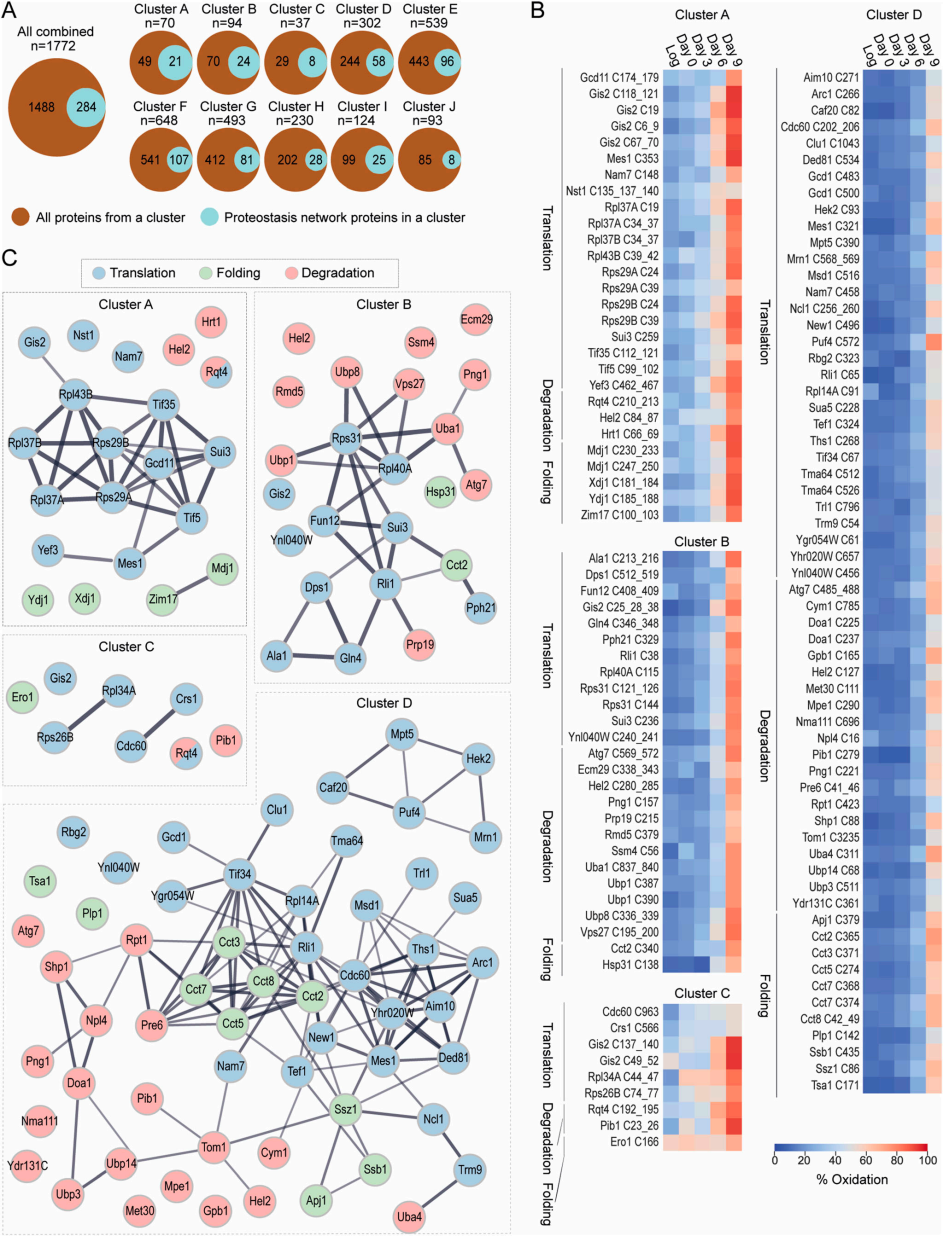
Proteins regulating proteostasis exhibit progressive oxidation patterns. **(A)** Venn diagram of the number of proteins involved in proteostasis (turquoise) among all proteins that have at least one Cys-peptide grouped within individual clusters (brown). Of 1,772 proteins, 16% of all quantified proteins regulate global proteostasis. For particular clusters, proteins involved in proteostasis account for 30% (cluster A), 25.5% (cluster B), 21.6% (cluster C), 19.2% (cluster D), 17.8% (cluster E), 16.5% (cluster F), 16.4 (cluster G), 12.2% (cluster H), 20.2 (cluster I), and 8.6% (cluster J). *n*, total number of all proteins within the cluster. **(B)** Heatmaps present the average % oxidation of Cys-peptides of proteostasis-regulating proteins from early- and mid-oxidized clusters A–D. Each Cys-peptide is presented in the form of cysteine ID (CysID): name of the protein followed by the residue abbreviation (cysteine, C) and position/s of the quantified residue/s within the peptide. Peptides are grouped in alphabetical order and by the biological process related to proteostasis (translation, degradation, folding). For proteins that belong to more than one process, a representative process is selected. *Log*, logarithmic phase. **(C)** STRING protein association network for proteostasis-regulating proteins for individual clusters A–D. Nodes, protein names; lines, interaction evidence. Line thickness, the strength of the evidence. Nodes are coloured based on the biological process the protein is involved in. Proteins involved in more than one process have more than one colour.

**Figure S5.**
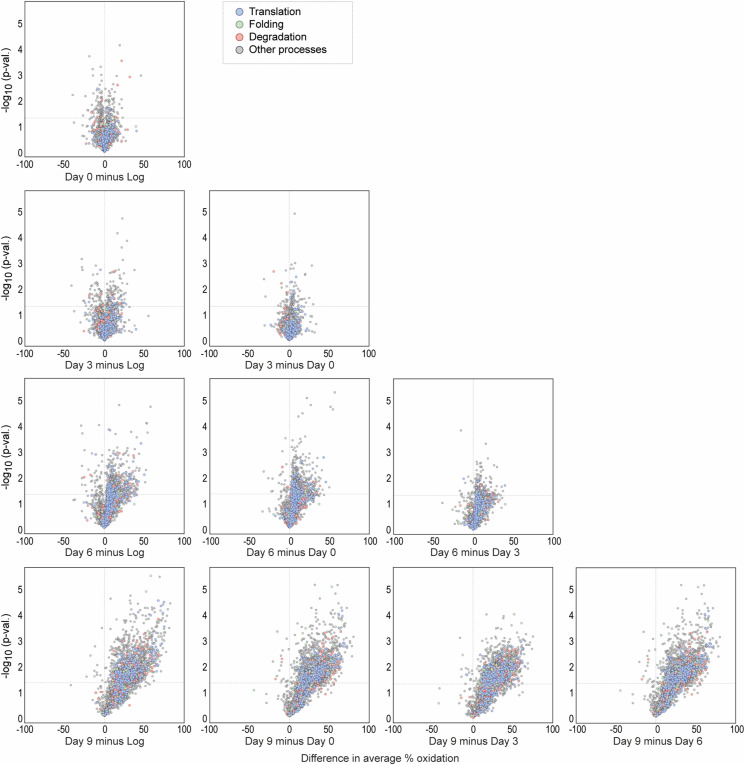
(Related to Fig 5)—Pairwise comparison of oxidation of cysteine residues of proteostasis-regulating proteins during ageing. Pairwise comparison of in vivo cysteine oxidation during logarithmic phase (Log) and days 0–9 of chronological ageing from the “yeast OxiAge” dataset (with imputed values) for proteins involved in proteostasis regulation. The difference in average % oxidation between time points from at least two biological replicates is shown on the *x*-axis; −log_10_ *P*-value (two-tailed Welch’s *t* test) is shown on the *y*-axis. Grey horizontal line, *P*-value = 0.05. Grey dots, peptides of proteins that were not classified to proteostasis processes; pastel blue dots, peptides of proteins regulating cytoplasmic translation; pastel green dots, peptides of proteins regulating protein folding; pastel red dots, peptides of proteins regulating protein degradation. *n*, number of Cys-peptides.

Protein homeostasis (proteostasis) depends on the coordinated interplay of protein production, folding, and degradation. Loss of proteostasis is a long-standing determinant of the ageing process (Kaushik & Cuervo, 2015; Hohn et al, 2017). Analysis of GO terms associated with proteostasis demonstrated the strongest overrepresentation of these processes in cluster A of early-oxidized proteins, with the highest enrichment for cytoplasmic translational initiation (fold enrichment > 12; corr. *P*-val. = 0.009, Fisher’s test) and protein refolding (fold enrichment > 8; corr. *P*-val. < 0.03, Fisher’s test) (Fig 4D). Moreover, early-oxidized proteins in cluster B were found to be significantly enriched in protein ubiquitination and degradation terms (fold enrichment > 2.5; corr. *P*-val. < 0.05, Fisher’s test). Because our functional analysis revealed that age-dependent oxidation might play a role in all three processes, we wanted to further determine the identity of the associated redox-sensitive proteins. We performed a search of GO terms and assigned all proteins to three groups: cytosolic translation, protein degradation, and protein folding (see the Materials and Methods section). In total, ∼16% of all proteins were annotated as involved in proteostasis (Fig 5A, Table S5). Cys-peptides of these proteins exhibited a similar oxidative shift towards higher values as cells progressed through ageing compared with peptides belonging to other processes. Notably, a strong preference for increased oxidation during early ageing was observed (Fig S5). Although the total number of proteostasis-regulating proteins was higher in bigger clusters (E–H), a higher percentage was associated with the smaller clusters A–C (Fig 5A). We focused our analysis on proteins within clusters A–D, which contained proteins with Cys-peptides oxidized during the early phase (clusters A–C) and mid-point (cluster D) of ageing. Cys-peptides within the clusters were modified at a similar level during the analysed time points. The oxidation levels of Cys-peptides were independent of the involvement of the protein in a particular proteostasis-associated process (Fig 5B). In cluster A, most of the proteostasis-regulating proteins were functionally related (Fig 5C). Here, we distinguished two main groups: (i) cytosolic ribosomal proteins and ribosome-associated proteins, and (ii) proteins involved in protein folding. Most striking was the enrichment in core ribosomal proteins including Rpl37A/B, Rpl43B, and Rps29A/B, as well as other translation-regulating proteins, such as Gis2, Yef3, Tif5, and Sui3. All these proteins were previously identified as redox-sensitive in response to exogenous oxidative stress in different species (Brandes et al, 2011; Menger et al, 2015; Topf et al, 2018; Su et al, 2019). In addition, we identified subunits of translation initiation factors, such as Tif35 and Gcd11, and proteins involved in degradation, namely, Rqt4, Hrt1, and Hel2. Rqt4 and Hel2 are both part of the ribosome-associated quality control triggering complex (RQT). The complex acts during ribosome stalling and contributes to the dissociation of the ribosome (Matsuo et al, 2017, 2020). Furthermore, we identified four cochaperones that are necessary for the import of precursor proteins into the mitochondria, namely, Ydj1, Xdj1, Mdj1, and Zim17. Although Mdj1 and Zim17 are localized in the mitochondrial matrix (Burri et al, 2004; D’Silva et al, 2003), Ydj1 and Xdj1 are cytosolic proteins that were found to deliver mitochondrial precursor proteins to the organelle (Xie et al, 2017; Opalinski et al, 2018).

Table S5 List of proteins from the “yeast OxiAge” dataset assigned to one of three main proteostasis-regulating processes: cytoplasmic translation, protein folding, or protein degradation.

Consistent with the GO enrichment analysis (Fig 3A), proteins with cysteine residues grouped within cluster B are mostly associated with protein degradation. Thus, the centre of the network is formed by ubiquitin-containing ribosomal proteins Rps31 and Rpl40A, with branches extending to proteins involved in translation and degradation (Fig 5C). About half of the proteins of proteostasis-regulating proteins within cluster B are associated with protein degradation. This includes the ubiquitin-specific proteases Ubp1 and Ubp8, the ubiquitin-activating enzyme Uba1, and E3 ubiquitin ligases Hel2 and Ssm4. Previous studies demonstrate that many enzymes involved in the UPS are sensitive to oxidative stress and oxidation of cysteine residues in the catalytic domains inactivates the enzymes either by changing the protein’s structure or by blocking the ubiquitin-binding site (Shang & Taylor, 2011). Within Hel2, a RING finger ubiquitin ligase (E3) and a component of the ribosome-associated quality control trigger complex, we identified several cysteine sites exhibiting oxidation during early ageing. Among them were Cys-sites within the catalytic RING domain (Cys79, Cys84, Cys87). Although we did not identify Cys64 and Cys67 of the Hel2 RING domain, their importance for ligase activity was demonstrated previously (Ikeuchi et al, 2019). Similarly, mutation of redox-sensitive Cys379 of Rmd5 ubiquitin ligase (cluster B) has been shown to impede the ubiquitination of its targets (Santt et al, 2008). Comparable findings extend to other E3 ligases and ubiquitin-specific proteases, including Cys878 in Hul5 (Crosas et al, 2006), Cys214 in Ubp15 (Kostetsky & Vladimirova, 1990), and Cys3235 in Tom1 (Saleh et al, 1998; Utsugi et al, 1999; Duncan et al, 2000). Moreover, blocking redox-sensitive cysteine sites in Png1 (N-glycanase) and Uba4 (E1-like protein) has also been observed to abolish the enzymatic activity of these proteins (Furukawa et al, 2000; Katiyar et al, 2002; Schmitz et al, 2008; Leidel et al, 2009). Importantly, the redox-regulated activity of deubiquitinating enzymes of the cysteine protease family contributes to the balance of ubiquitinated protein species in the cell. Yuh1 is an example of the DUB enzyme that contains cysteine sites oxidized during ageing that reside within the active site of the protein (Rajesh et al, 1999; Sakamoto et al, 1999; Kanamori et al, 2011).

Besides proteins involved in the UPS, several proteins with Cys-peptides found in cluster B were involved in cytoplasmic translation. However, in contrast to cluster A, only a few were located within the ribosome itself (Table S3; Peptides and Localization). Among the proteins involved in the translation process were the translation initiation factor Sui3, tRNA synthetases (Dps1, Ala1, Gln4), and proteins involved in ribosome biogenesis (Fun12, Rli1). It is largely unknown how these factors are regulated by oxidative thiol modification. It was shown that oxidation of tRNA synthetases impairs editing activity and consequently decreases translation fidelity because of the incorporation of wrong amino acids during polypeptide chain production (Ling & Soll, 2010).

Balance in protein homeostasis during ageing is also regulated by folding and refolding of proteins. Some of the cysteine sites of proteins involved in the control of folding, and oxidized during early and mid-point of ageing, were found previously to be required for the proper functioning of these processes. For example, we found that Cys138 within Hsp31 exhibits early oxidation during ageing (cluster B). This residue resides within the active site of the protein and is known for its susceptibility to oxidation (Graille et al, 2004; Wilson et al, 2004).

Six proteins of cluster C were involved in cytosolic translation. Among them were two ribosomal proteins, Rps26B and Rpl34A, and two tRNA synthetases, Cdc60 and Crs1/YNL247W (Fig 5C). Both ribosomal proteins were found to have a single peptide oxidized on average between 20% and 30% with an increase up to even 60% on day 0, stabilizing at this level until day 6 (Fig 5B). Both peptides were also found to be significantly more oxidized in proliferating cells in response to exogenous and endogenous oxidative stress (Topf et al, 2018).

Cluster D contained a total of 29 proteins involved in translation, with only one ribosomal protein, Rpl14A. Other translation-associated proteins formed subclusters including tRNA synthetases (e.g., Cdc60, Msd1, Ths1, Mes1, Ded81), regulators of tRNA function (e.g., Sua5, Ncl1, Trm9, Arc1), mRNA-binding proteins (e.g., Caf20, Puf4, Mrn1, Hek2), and translation initiation factors (e.g., Tif34, Clu1) (Fig 5C). The second most noticeable group of proteins is related to protein folding, specifically de novo folding downstream of translation, and includes the chaperonin complex TRiC/CCT. This complex consists of eight subunits. We identified five of them within cluster D: Cct2, Cct3, Cct5, Cct7, and Cct8. Moreover, Cys-peptides of the ribosome-associated chaperones Ssz1 and Ssb1 were quantified. This might indicate that the protein folding machinery is a target of redox regulation during the mid-point of ageing. Other proteins with Cys-peptides in cluster D were described previously to be oxidized. Among them, the thioredoxin peroxidase Tsa1 displayed oxidation at a single cysteine site, Cys171. This site is the resolving cysteine in the peroxidatic cycle forming a disulphide bond with Cys48 in the active site of Tsa1 (Chae et al, 1994a, 1994b).

**Figure S6.**
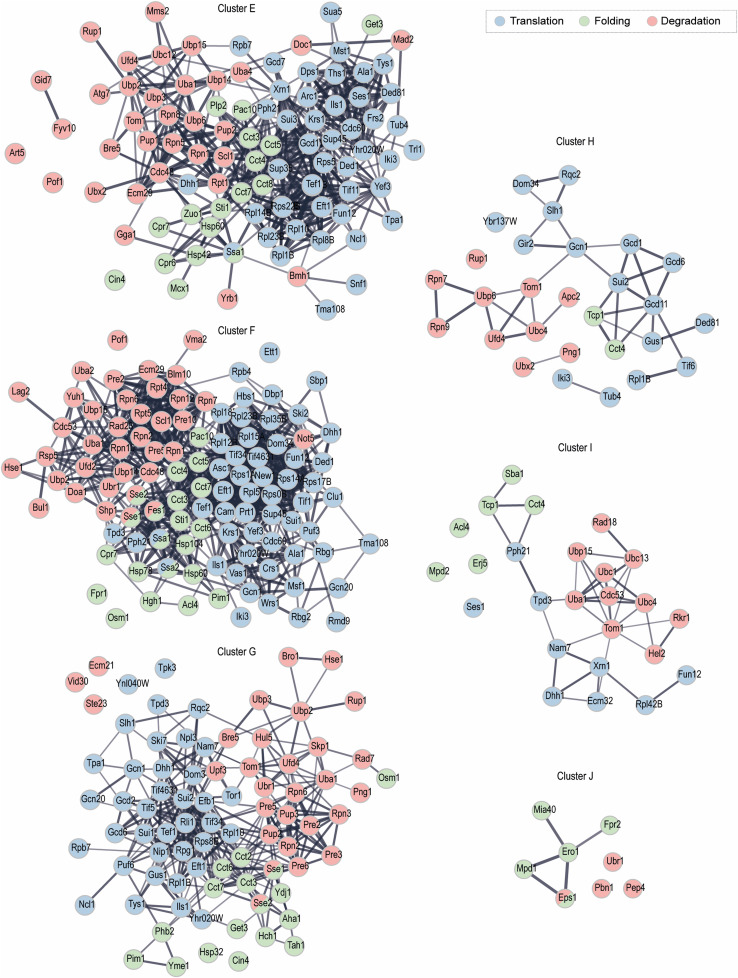
(Related to Fig 5)—Protein association networks for proteostasis-regulating proteins within clusters E–J. STRING protein association network for proteostasis-regulating proteins for individual clusters E–J. Nodes, protein names; lines, interaction evidence; line thickness, the strength of the evidence. Nodes are coloured based on the biological process the protein is involved in. Proteins involved in more than one process have more than one colour.

Within cluster D, 19 proteins were involved in degradation forming a distinct but dispersed group (Fig 5C). The larger subgroup belongs to enzymes of the UPS involved in ubiquitin ligation (E1; e.g., Tom1, Hel2, Pib1), ubiquitin removal (DUBs; Ubp14, Ubp3), and ubiquitin chain–binding proteins (Npl4, Shp1). Moreover, the proteasome subunits Rpt1 (ATPase of the regulatory particle) and Pre6 (alpha subunit of the core) were identified. However, most proteasome subunits had Cys-peptides grouped in clusters F and G (Fig S6), which suggests that the proteasome is a target of oxidation only at the late stages of ageing.

### Early oxidation of proteostasis-regulating proteins is conserved among species

**Figure 6.**
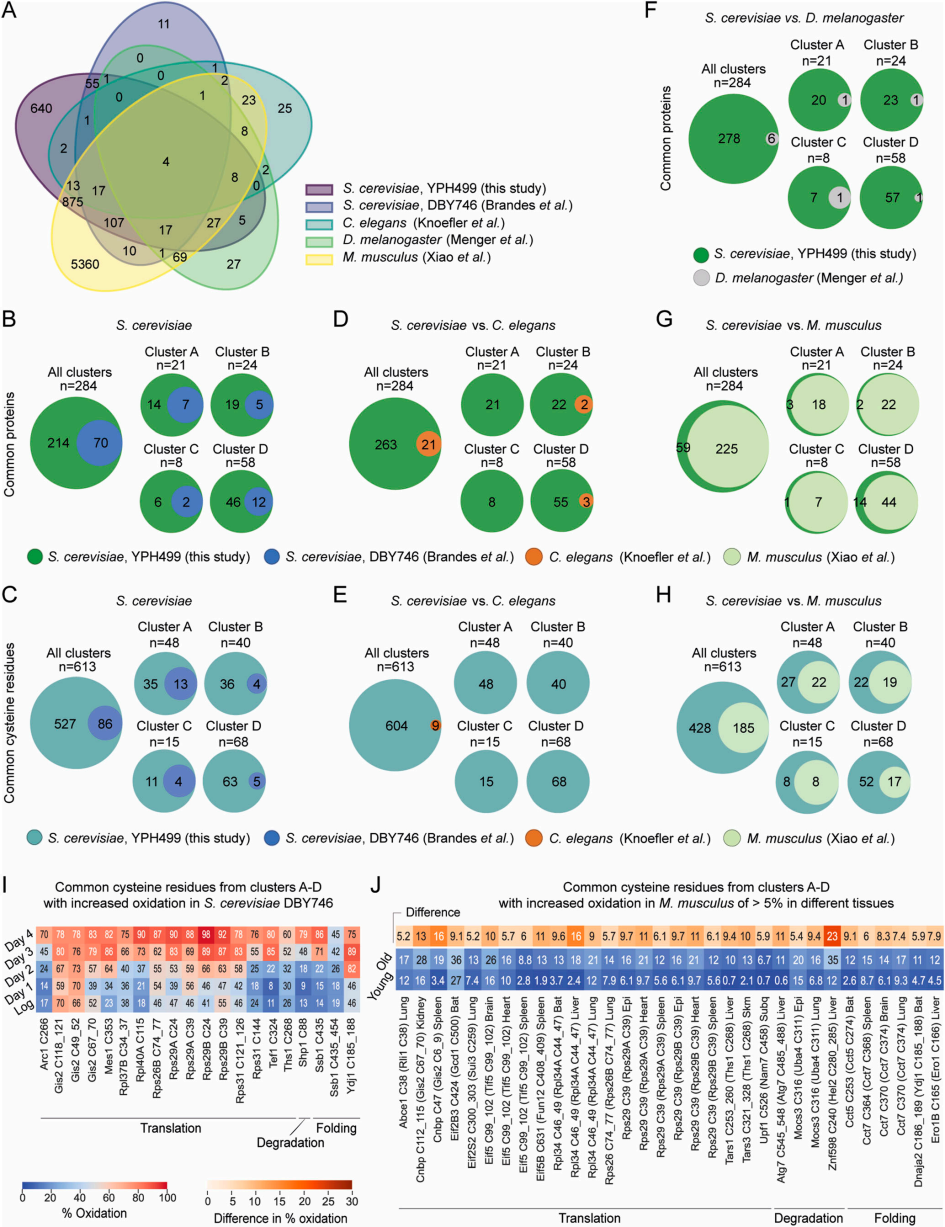
Evolutionary conservation of thiol oxidation of proteostasis-regulating proteins during ageing. **(A)** Venn diagram of proteins commonly found as reversibly oxidized during ageing in different species. **(B, C, D, E, F, G, H)** Pairwise comparison of commonly oxidized proteins (B, D, F, G) and cysteine residues (C, E, H) between species. Orthologs were compared with the whole “yeast OxiAge” dataset (“All clusters”) and each cluster A–D individually. Green, unique proteins from the “yeast OxiAge” dataset (B, D, F, G); turquoise, unique cysteine residues from the “yeast OxiAge” dataset (C, E, H); blue, unique proteins or cysteine residues from the yeast Brandes et al dataset (Brandes et al, 2013); brown, unique proteins or cysteine residues from the worm Knoefler et al dataset (Knoefler et al, 2012); grey, unique proteins from the fruit fly Menger et al dataset (Menger et al, 2015); pale green, unique proteins or cysteine residues from the mouse Xiao et al dataset (Xiao et al, 2020). **(I)** Heatmap of average % oxidation of Cys-peptides from the yeast Brandes et al dataset (Brandes et al, 2013) for commonly oxidized cysteine residues with the “yeast OxiAge” dataset. Only peptides from clusters A–D of “yeast OxiAge” and peptides with increased oxidation in Brandes et al (2013) are shown. Peptides are presented in alphabetical order and sorted based on the biological process. *Log*, logarithmic phase. **(J)** Heatmap of average % oxidation of Cys-peptides from the Xiao et al dataset (Xiao et al, 2020) for commonly oxidized cysteine residues with the “yeast OxiAge” dataset. Only peptides from clusters A–D of “yeast OxiAge” and peptides with increased oxidation of >5% in Xiao et al (2020) are shown. Peptides are presented in alphabetical order and sorted based on the biological process. The difference in average % oxidation between old and young mice is shown in a single-colour warm palette heatmap.

The increase in ROS accompanied by increasing oxidative damage of proteins during ageing is a universal phenomenon also found in higher eukaryotes (Agarwal & Sohal, 1996; Stadtman, 2001; Hohn et al, 2013; Gladyshev et al, 2021). To the best of our knowledge, reversible, oxidative modifications during organismal ageing were reported at a proteome-wide scale only in four other datasets: budding yeast *S. cerevisiae* WT strain DBY746 (Brandes et al, 2013), nematode *C. elegans* WT strain N2 (Knoefler et al, 2012), fruit fly *D. melanogaster* WT strain *white Dahomey* (Menger et al, 2015), and mouse *Mus musculus* WT strain C57BL/6 (Xiao et al, 2020). We compared the published datasets (Table S6) with the “yeast OxiAge” compendium for commonly identified proteins with reactive thiol groups (Table S2). However, the analysis was limited by the different methodologies used and the total number of proteins identified in the different studies. Thus, the overlap between all the datasets was rather small (Fig 6A and Table S7). We reasoned that most likely early-oxidized proteins would have the largest impact on cells’ functions during ageing. Because the decline in proteostasis is a universal hallmark of ageing (Lopez-Otin et al, 2023), we specifically searched in the published datasets for the equivalent proteins and cysteine residues that we identified in the “yeast OxiAge” sub-dataset for proteostasis (Tables S8 and S9). We speculated that in other organisms similar functional classes of proteins might coordinate the ageing process. Therefore, we pairwise matched only the proteostasis-regulating proteins quantified in the “yeast OxiAge” dataset with the four existing quantitative redoxome datasets of aged eukaryotes.

Table S6 List of filtered Cys-peptides from the *S. cerevisiae* Brandes et al (2013) dataset, *C. elegans* Knoefler et al (2012) dataset, *D. melanogaster* Menger et al (2015) dataset, and *M. musculus* Xiao et al (2020) dataset.

Table S7 List of all proteins commonly oxidized during ageing between four species and five datasets: yeast *S. cerevisiae*, strain YPH499 from this study (“yeast OxiAge”), yeast *S. cerevisiae*, strain DBY746 from the Brandes et al (2013) dataset, nematode *C. elegans* from the Knoefler et al (2012) dataset, *D. melanogaster* from the Menger et al (2015) dataset, and mouse *M. musculus* from the Xiao et al (2020) dataset.

Table S8 List of proteins commonly oxidized during ageing for the “yeast OxiAge” proteostasis sub-dataset and the Brandes et al (2013), Knoefler et al (2012), Menger et al (2015), and Xiao et al (2020) datasets, pairwise comparison.

Table S9 List of cysteine residues commonly oxidized during ageing for the “yeast OxiAge” proteostasis sub-dataset and the Brandes et al (2013), Knoefler et al (2012), Menger et al (2015), and Xiao et al (2020) datasets, pairwise comparison.

**Figure S7.**
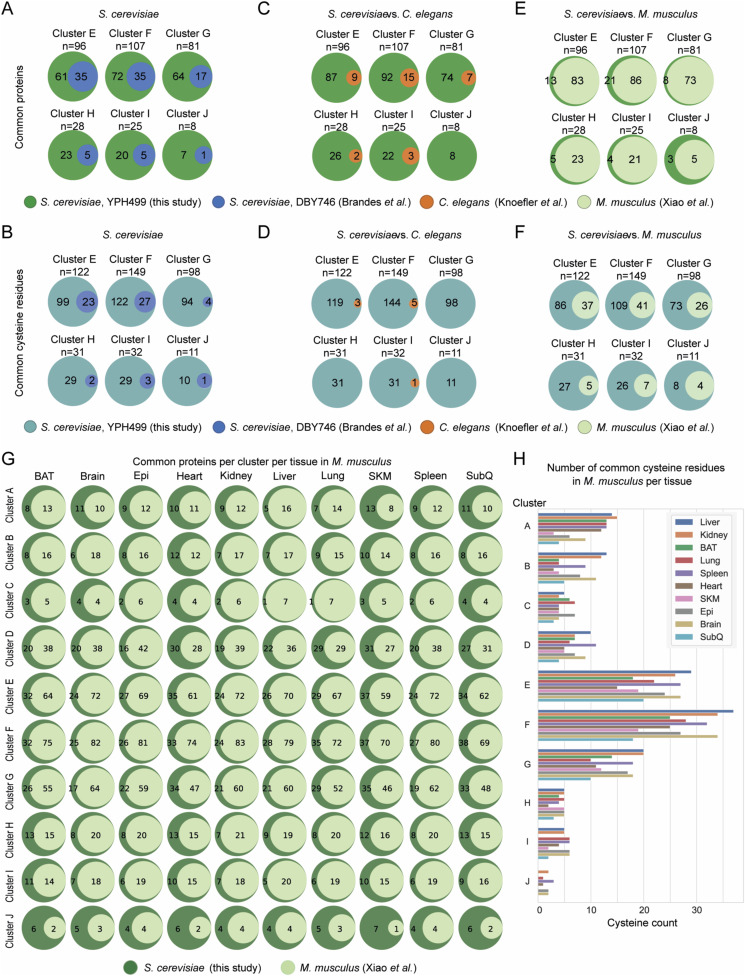
(Related to Fig 6)—Cross-species comparison of oxidation of proteostasis-regulating proteins during ageing. **(A, B, C, D, E, F)** Pairwise comparison of commonly oxidized proteins (A, C, E) and cysteine residues (B, D, F) between species. Orthologs were compared with each cluster E–J individually. Green, unique proteins from the “yeast OxiAge” dataset (A, C, E); turquoise, unique cysteine residues from the “yeast OxiAge” dataset (B, D, F); blue, unique proteins or cysteine residues from the yeast Brandes et al dataset (Brandes et al, 2013); brown, unique proteins or cysteine residues from the worm Knoefler et al dataset (Knoefler et al, 2012); pale green, unique proteins or cysteine residues from the mouse Xiao et al dataset (Xiao et al, 2020). **(G)** Pairwise comparison of commonly oxidized proteins between the “yeast OxiAge” dataset and the Xiao et al dataset (Xiao et al, 2020) for individual clusters and mouse tissues. Green, unique proteins from the “yeast OxiAge” dataset; pale green, unique proteins from the Xiao et al (2020) dataset. *BAT*, brown adipose tissue; *Epi*, epididymal fat; *SLM*, skeletal muscle; *SubQ*, subcutaneous fat. **(H)** Number of common cysteine residues between the “yeast OxiAge” dataset and the mouse Xiao et al dataset (Xiao et al, 2020) per each tissue per cluster. *BAT*, brown adipose tissue; *Epi*, epididymal fat; *SLM*, skeletal muscle; *SubQ*, subcutaneous fat.

First, we compared the two yeast strains: YPH499 (this study, “yeast OxiAge”) and DBY746 (Brandes et al, 2013). We identified 70 proteins involved in proteostasis as redox-sensitive (Fig 6B) with 86 common cysteine residues (Fig 6C). As expected, the highest number of matching proteins and cysteine residues was present in the largest clusters of the late oxidation, clusters E and F (Fig S7A and B, Tables S8 and S9). For clusters A-D with proteins showing early- and mid-point increase in oxidation, 26 proteins were identified in both yeast strains (Fig 6B). Among them were ribosomal proteins, such as Rpl37B, Rps26B, or Rps31, regulators of translation, such as Yef3, Tef1, and Gis2, and tRNA synthetases, that is, Mes1 and Dps1, and proteins involved in folding, that is, CCT components and Ydj1 (Fig 6I, Table S8). Furthermore, 36 specific cysteine residues were quantified in both yeast datasets for proteostasis-regulating proteins from clusters A–D (Fig 6C). Three of 26 proteins did not have any matching quantified cysteine residue, including Cct8 (“yeast OxiAge” Cys-sites 42, 49, 252, and 298 and Brandes et al [2013] Cys336), Gcd1 (“yeast OxiAge” Cys-sites 356, 370, 483, and 500 and Brandes et al [2013] Cys172), and Ssz1 (“yeast OxiAge” Cys-sites 86 and Brandes et al [2013] Cys81). Commonly identified cysteine residues that grouped in clusters A–D and exhibit an increase in oxidation during ageing in both datasets are shown in Fig 6I.

Next, we compared proteins with reactive thiols between different species. We mapped redox-sensitive proteins oxidized during different phases of ageing in WT nematode *C. elegans* (Knoefler et al, 2012), WT fruit flies (Menger et al, 2015), and young and old mice (Xiao et al, 2020) to the “yeast OxiAge” dataset. We used ∼130 proteins from the worm dataset after filtering (see the Materials and Methods section) with identified oxidation states. We found 21 proteostasis-regulating proteins common with the yeast orthologs oxidized in our proteostasis subset (Figs 6D and S7C). The small list of common proteins included four found in clusters A–D, namely, Eft-3 (Sc_Tef1), Dars-1 (Sc_ Dps1), Cct8, and Cct2 (two peptides found in different clusters, B and D) (Fig 6D). Oxidation of nine evolutionary conserved cysteine residues between yeast and *C. elegans* was identified, but none of them belonged to the early-oxidation targets grouped in clusters A–D (Figs 6E and S7D, Table S8). Similarly, the rather small fruit fly dataset (121 proteins and 252 cysteine residues after filtering; see the Materials and Methods section) contained only two proteins that were found early-oxidized in the “OxiAge” dataset: CNBP (Sc_Gis2) and Prx1/2/4 (Sc_Tsa1) (Fig 6F). In total, six common proteins were identified across different clusters with no commonly oxidized cysteine residue (Table S8).

A larger number of proteostasis-regulating proteins were commonly found in mice and yeast. On average, ∼50% of yeast proteins had homologs quantified in the mouse study (Figs 6G and S7E, Table S8). Among proteins oxidized during yeast early and mid-point of ageing (clusters A–D), the highest proportion of common proteins (∼65%) was seen in cluster B (Fig 6G). The mouse study contained data obtained from only two time points: young (16-wk-old) and old (80-wk-old) animals. However, it allowed for unique tissue-specific identification of redox-sensitive proteins. We observed that the percentage of quantified proteins common with yeast proteostasis sub-dataset is comparable between tissues (Fig S7G). The highest number of common early-oxidized proteins (clusters A–C) is found in the brain tissue (19 proteins, >75% of cluster B), followed by the brown adipose tissue, epididymal fat (epi), kidney, liver, subcutaneous fat (subQ), and spleen (17 proteins each, >65% of cluster B). We identified 185 evolutionary conserved redox-sensitive cysteine sites between mice and yeast belonging to proteins within the proteostasis sub-dataset (Figs 6H and S7F). Among them, 66 were within the early-oxidation targets in cluster A–D. The total number of common cysteine residues was spread unevenly between tissues (Fig S7H, Table S9). Clusters A and D exhibited the smallest number of common cysteine residues quantified in subQ and liver, respectively, whereas the highest number of 12 was identified in the brown adipose tissue for early-oxidized cysteine residues (cluster A). For 16 proteins with Cys-peptides from clusters A–D in different tissues, we observed an increase in oxidation of more than 5% between young and old mice (Fig 6J), whereas for an additional 15 proteins, we observed an increase in oxidation of less than 5% (Table S8). Among the proteins with evolutionarily conserved cysteine residues showing an age-dependent increase in oxidation in mice were proteins involved in translation. This includes ribosomal proteins (e.g., Rpl34 [Sc_Rpl34A/B], Rps26 [Sc_Rps26B]), ribosome-associated factors (e.g., Eif2S3 [Sc_Gdc1], Eif2S2 [Sc_Sui3], Eif5 [Sc_Tif5], Cnbp [Sc_Gis2]), and tRNA synthetases (e.g., Tars1 [Sc_Ths1]). In addition, common proteins with increased % oxidation during ageing included the ubiquitin ligase Znf598 (Sc_Hel2), ubiquitin protease Mocs3 (Sc_Uba4), and components of the TRiC/CCT chaperone complex (e.g., Cct5 [Sc_Cct5], Cct7 [Sc_Cct7]]) and cytosolic cochaperone Dnaja2 (Sc_Ydj1) (Fig 6J).

The integration of the newly described “yeast OxiAge” dataset with existing datasets in yeast, worms, fruit flies, and mice provides the opportunity to identify evolutionarily conserved cysteine residues that are redox-sensitive and to study the dynamics of oxidative modification during ageing. Furthermore, it allows the targeted analysis of proteins and cysteine residues that were not experimentally identified in each organism, and in this respect, the existing datasets complement the “yeast OxiAge” dataset.

### An online tool that allows easy search and visualization of cross-species comparison of thiol–proteome oxidation during ageing

**Figure 7.**
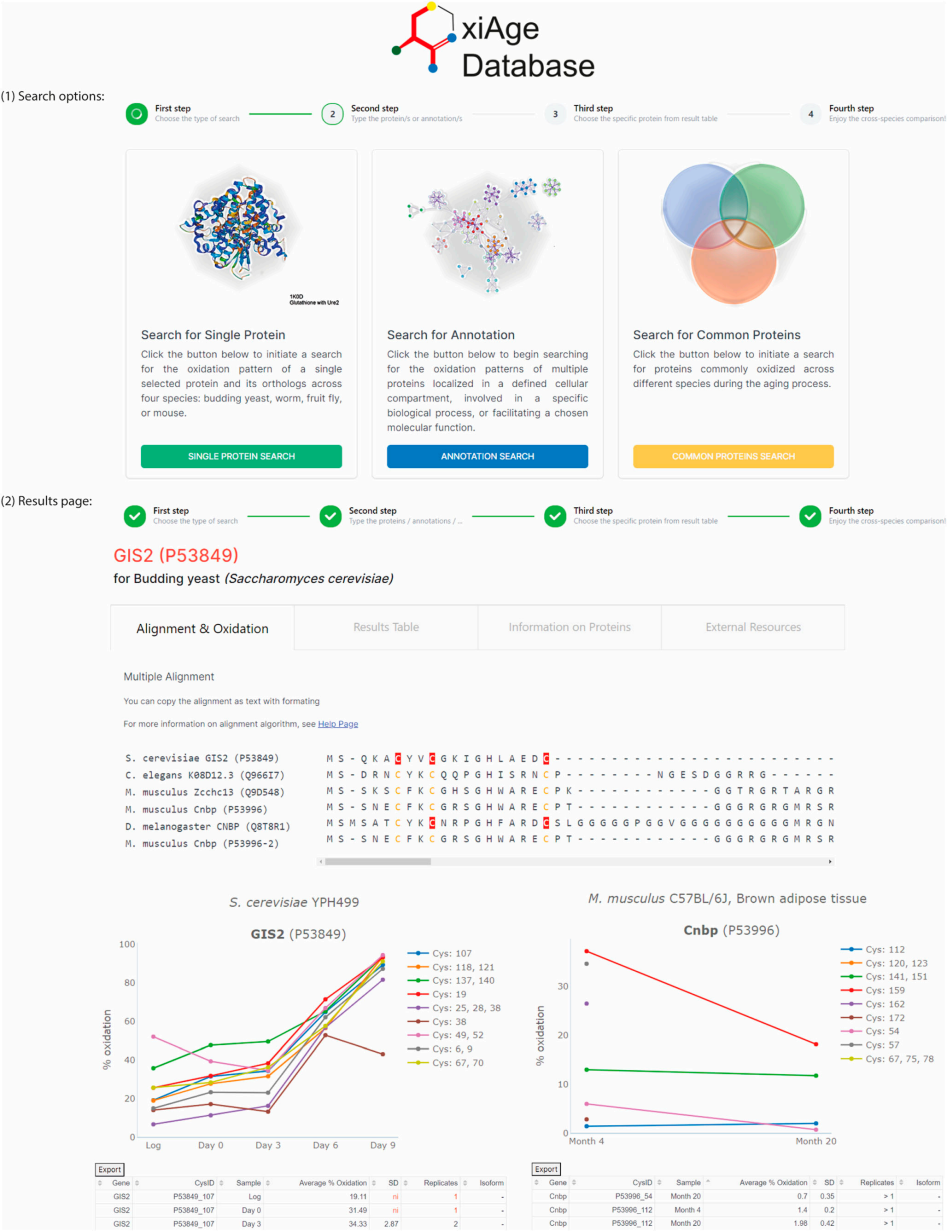
Design of the OxiAge web application. Screenshots of OxiAge Database web application. (1) Search options: the user can choose three options for searching the database: for individual protein/gene, for multiple redox-sensitive proteins common between different species, or for proteins annotated in the GO term of chosen biological process, molecular function, or cellular component. (2) Results page: the final output of the search presents detailed information about cysteine residues of multiple species found oxidized during ageing. The final results are presented in the form of four tabs. Within the first tab, amino acid alignment is shown with detected cysteines marked in red (information on cysteine position is visible in a tooltip). Graphs present the average % oxidation of cysteines within each peptide. Each found protein and species has a separate graph. Graphs for multiple mouse tissues are presented at the bottom of the tab. Details about the particular experiment and tables containing information about oxidation are shown next to each graph. The second tab contains a table with collected oxidation data for all species and experiments from the first tab. Within the third tab, detailed information on chosen proteins and orthologs is found along with the table containing motifs surrounding all cysteine residues. Within the fourth tab, links to external resources for chosen proteins and orthologs are found.

**Figure S8.**
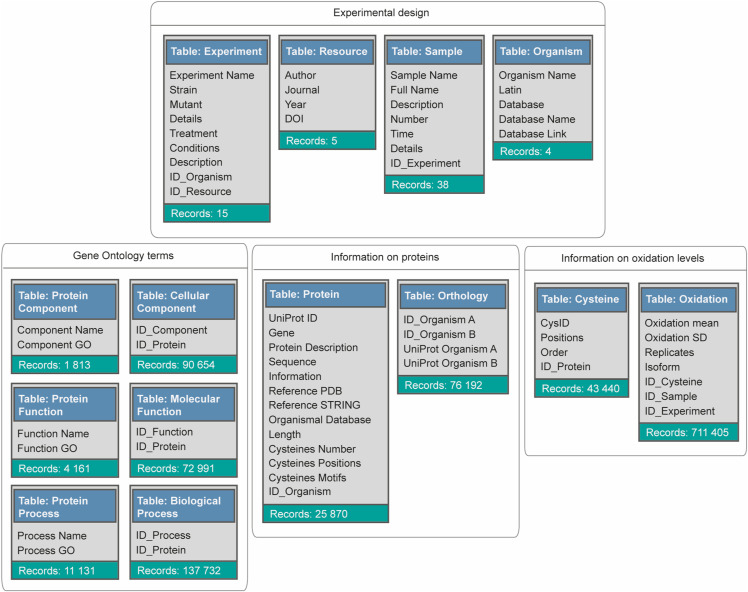
(Related to Fig 7)—Design of the OxiAge Database. Design of the MySQL database containing information about the average % oxidation of aged proteome of *S. cerevisiae* (this study and Brandes et al [2013]), *C. elegans* (Knoefler et al, 2012), *D. melanogaster* (Menger et al, 2015), and *M. musculus* (Xiao et al, 2020). Unfiltered data were used to allow the user to perform custom filtering based on individual criteria. The database consists of 14 main tables divided into four groups (grey squares). Schemes of the tables contain the number of records (rows) within a table (sea-green background), and column names within each table. Column names starting with “ID” indicate the identification number referencing a record from another table.

To provide an easily accessible interphase for the research community, we developed the first cross-species platform, called OxiAge Database, that can be accessed through a web application (http://oxiage.ibb.waw.pl; Fig 7). The database contains information on the oxidation levels of ageing yeast (this work and Brandes et al [2013]), worms (Knoefler et al, 2012), fruit flies (Menger et al, 2015), and mice (Xiao et al, 2020). Our compendium contains information on the oxidation of 13,051 proteins in canonical or isoform forms (3,153 in yeast, 138 in worms, 420 in flies, 9,340 in mice) and a total of 43,440 cysteine-containing peptides. Each peptide of currently available UniProt ID and detected in any of the datasets has a record in the database. The results are unfiltered, allowing the users to filter according to their interests. In addition, the user can download the filtered “yeast OxiAge” dataset with the basic analysis presented in this work. The number of records containing information about oxidation levels at different time points/samples is above 711,000 (Fig S8). The web application allows for comparison between proteins with detected cysteine residues and proteins that were not found in any of the datasets, but their genes are known orthologs of the detected ones. Thus, the total number of all proteins included in the database reaches almost 26,000 for four different species. The number of ortholog pairs between them is almost 76,200.

The database allows three distinct types of searches (Fig 7), including searching for a single specified protein within a chosen organism or searching for multiple proteins within a specific biological process, molecular function, or cellular location (according to Gene Ontology annotations). Furthermore, the user can search for all proteins commonly oxidized between two, three, or four chosen species. The database is designed to allow easy adjustments for new search procedures and the insertion of additional datasets or information from the existing records (Fig S8).

During the search, the user receives information about the searched protein and the orthologs found in other species, along with the sequence alignment with marked cysteine residues detected in a particular organism (Fig 7). In addition, the user receives results of multiple alignments between proteins with detected cysteine oxidation and orthologs that were not detected within the analysed datasets. Such comparison may serve as a prediction tool for thiol oxidation of proteins that were not found in any of the ageing datasets but may be potential targets of oxidation. The graphs of oxidation levels over time allow for quick determination of the oxidation change for a particular organism and mouse tissue (Fig 7). Users can download information about orthologs and protein oxidation. In addition, a table with all positions of cysteine residues found within a set of proteins, as well as surrounding motifs, is supplied. The results tabs present additional information about searched proteins and orthologs, among them direct links to external resources, such as organism-specific databases (*Saccharomyces* Genome Database, SGD; WormBase: Nematode Information Resource, WB; FlyBase, FB; Mouse Genome Informatics, MGI), STRING interaction networks, and predicted protein structure by AlphaFold (Jumper et al, 2021) or solved crystal structures from Protein Data Bank.

## Discussion

We present a comprehensive dataset on age-dependent protein thiol modifications, providing a rich resource for the research community. The freely available online tool, “OxiAge” Database, which enables cross-species comparison of the conservation of oxidation-sensitive cysteine residues, accompanies the dataset. Different search modes not only enable a targeted approach but also allow for exploring open-ended scientific questions. The database and the web application are designed to allow for the integration of new datasets that might be published in future, as well as the addition of new search and display functions.

The advantage of the “yeast OxiAge” dataset is the temporal resolution of oxidation of 3,178 cysteine residue–containing peptides. A high number of quantified Cys-peptides enable the unbiased search for early-oxidation targets during ageing. Although most quantified Cys-peptides have a single cysteine, enabling precise identification of ageing effects, early-oxidized peptides with multiple cysteine residues pose a challenge in interpreting the obtained data. Further experimental analysis will be necessary to delineate the contribution of % oxidation of multiple cysteine residues within the same peptide.

The functions of cysteine residues within a protein are diverse, encompassing roles in structural stability and protein folding through the creation of disulphide bonds, participation in enzymatic activity and redox reactions within active sites, and engagement in signal transduction and redox sensing through post-translational modifications, such as phosphorylation and nitrosylation. Specific cysteine residues may be more susceptible to oxidation, influenced by factors such as microenvironment and local structural features. Cysteine residues in close proximity may have a higher likelihood of forming disulphide bonds within the protein structure, rendering them more prone to oxidation. Disulphide bond formation can support the catalytic function of proteins, for example, shown for Tsa1 and Grx1 (Chae et al, 1994a; Yu et al, 2008). In the case of other proteins that contain disulphide bonds, they may play a structural role. Exg1 and She10 are examples of proteins with redox-sensitive cysteine residues, which were not described as involved in a catalytic activity. The sensitivity of cysteine residues may also be influenced by their proximity to amino acids other than cysteine. The interaction between cysteine residues and positively charged amino acids entails an attraction between the negatively charged thiolates of cysteine and the positively charged side groups of other amino acids, such as arginine. This interaction can modulate the access or binding of ions and substrates to the protein, acting as a form of electrostatic gating (Xiao et al, 2020). Furthermore, the interaction between a cysteine residue and aromatic amino acids may influence conformational changes in proteins, affecting their function and stability. For example, in alpha-helixes, cysteine residues can be often found. Cdc60 and Crs1/YNL247W are two tRNA ligases that have redox-sensitive cysteine sites (cluster C) possibly interacting with aromatic amino acids (AF-P26637-F1 and AF-P53852-F1, AlphaFold [Jumper et al, 2021]). Thus, a detailed analysis of cysteine-containing motifs can help to predict the cysteine residue’s sensitivity to oxidative modifications.

Our analysis of protein oxidation during ageing uncovers a gradual increase in proteome oxidation that aligns with elevated cellular ROS levels, consistent with findings from a prior yeast study (Brandes et al, 2013). Notably, a gradual rise in thiol oxidation occurs until the mid-point of ageing, followed by a robust shift at day 9, which correlates with a significant increase in cellular ROS levels. This prompts questions about the factors driving this specific shift at day 9 and the subsequent decline in cell survival. We could not link changes in overall proteome oxidation to pH variations, glucose availability, or possible adaptive regrowth (Fabrizio & Longo, 2003). Instead, the intensified ROS suggested increased oxidative stress in the later stages of chronological ageing. ROS, natural by-products of cellular metabolism, rise with cell age, contributing to the observed increase in reversible oxidation of Cys-peptides. Alterations in the cellular redox balance or changes in nutrient availability during ageing could influence ROS production. Nutrient availability, especially through the TOR signalling pathway, intricately regulates mitochondrial function, influencing ROS and protein oxidation during chronological ageing (Loewith et al, 2002; Powers et al, 2006; Bonawitz et al, 2007; Pan, 2011). Increased respiration in a long-lived *tor1∆* mutant is crucial for yeast longevity, emphasizing the dependency on nutrient-modulated metabolic pathways and mitochondrial activity (Bonawitz et al, 2007). Elevated glucose levels contribute to increased ROS production (Roux et al, 2009). A study conducted by Brandes et al (2013) on the effect of caloric restriction and starvation on proteome oxidation in yeast demonstrated that reducing nutrients at the onset of chronological ageing or transitioning to starvation conditions in water after initial growth in standard 2% glucose conditions delayed thiol oxidation in yeast. Our analysis, performed under standard conditions, validates a faster increase in proteome oxidation compared to yeast cultures with reduced nutrients. Hence, the observed increase in oxidation cannot be attributed to nutrient depletion during the transition to the stationary growth phase.

In our study, we detected a 10 times higher number of oxidized Cys-peptides compared with the previous work within distinct phases of ageing (Brandes et al, 2013). We applied a clustering method of Cys-containing peptides that allowed the unravelling of an intricate hierarchy of oxidation events during yeast ageing. By dividing the Cys-peptides into 10 clusters, our approach not only categorized them based on general oxidation patterns but also considered the average % oxidation during the initial Log and early ageing. This nuanced grouping aimed to capture subtle differences in the oxidation profiles, revealing specific regulatory patterns in the associated proteins.

Our deliberate choice of a two-step clustering approach facilitated the identification of small differences at distinct time points, offering insights into the temporal dynamics of thiol oxidation. Hence, our analysis reveals a hierarchy of cellular responses affected by these reversible redox changes. At the earliest stage of chronological ageing, proteins involved in cellular oxidant detoxification and response to ROS are affected, which is consistent with the observation of a global increase in ROS upon entry into the stationary phase through a TOR-dependent pathway regulating mitochondrial respiration (Pan et al, 2011). These processes are represented by proteins already highly oxidized during the entry into the stationary phase and are mostly associated with cluster C. A further increase in the protein population with reversible oxidation of cysteine residues within cluster C during the first response to ageing (day 0) could indicate that they are involved in an active repair of thiol-oxidized proteins, linking their activity to the maintenance of cellular redox balance.

During the following days of early ageing, proteins involved in global proteome homeostasis through changes in protein production are becoming oxidized (cluster A). Particularly, we observed enrichment in proteins regulating ribosome assembly and cytosolic translation. Moreover, we detected changes in the oxidation of subunits of polymerase I required for the transcription of rRNA. Thus, cells may prioritize the regulation of global protein synthesis above other processes during the early stages of chronological ageing, which allows for quick adaptation to the increasing burden of misfolded/damaged proteins in the ageing cell. Regulation of translation is a common stress response, and reducing global protein production has been shown to extend lifespan in model organisms by targeting components of the translation machinery early in life (Anisimova et al, 2018; Chiocchetti et al, 2007; Curran & Ruvkun, 2007; Hansen et al, 2007; Rogers et al, 2011; Steffen et al, 2008; Tavernarakis, 2008; Topf et al, 2019; von der Haar et al, 2017). Prominent in our analysis was the effect on ribosomal proteins, which also was identified during stress conditions in previous studies (Marino et al, 2010; Kumsta et al, 2011; Menger et al, 2015; Topf et al, 2018). However, mechanistic consequences of the reversible oxidation of ribosomal proteins remain to be elucidated. Recently, it was shown that upon treatment of yeast cells with hydrogen peroxide cysteine residues of the ribosomal proteins, Rps26 and Rpl10 become readily oxidized leading to protein extraction from the ribosome and degradation (Yang et al, 2023). The authors suggest that subsequently oxidized proteins are replaced by newly synthesized Rps26 and Rpl10 resulting in ribosome repair upon oxidative stress. Whether similar mechanisms exist for oxidation-sensitive ribosomal proteins during ageing remains to be demonstrated. In addition, we identified Gis2, a translational activator for mRNAs with internal ribosome entry sites, to have several conserved cysteine residues sensitive to oxidation during the early stages of ageing. It was shown previously that Gis2 and its human homolog CNBP are associated with stress-induced RNP granules, suggesting a role in translational repression (Rojas et al, 2012). We also observed several tRNA synthetases among the targets of early oxidation. This confirms previous findings in mice that identified these proteins as the most ubiquitously oxidized between different tissues of aged animals in comparison with other proteins (Xiao et al, 2020).

The increased burden in damaged proteins through elevated levels of ROS in the ageing cell triggers further adaptation in cellular processes, including changes in the global transcription through regulation of polymerase II subunits and modifications in protein degradation machinery (cluster B). We found that redox-dependent regulation of protein degradation follows the adaptation of translation and transcription rather than being the first line of defence during ageing. The most pronounced effect was for the enzymes of the ubiquitination cycle. The sensitivity of the active site cysteine residues of ubiquitinating and deubiquitinating enzymes was shown previously. An example is an oxidized glutathione that was shown to react with the active site cysteine in E1 and E2 forming mixed disulphide bonds and thus blocking their binding to ubiquitin (Jahngen-Hodge et al, 1997). However, the activity of deubiquitinating enzymes (DUBs) is known to ensure a balance in the ubiquitinated proteome. DUBs are especially sensitive to oxidative stress. A single molecule, such as hydrogen peroxide, can affect a vast number of DUBs without the need for an enzymatic intermediate (Snyder & Silva, 2021). Furthermore, functional links between ubiquitination factors and translation were established (Kapadia & Gartenhaus, 2019). Protein synthesis can be controlled by the ubiquitination of ribosomes. Oxidative stress induces high levels of K63-linked polyubiquitin chains on ribosomes (Dougherty et al, 2020), and polyubiquitination pauses translation elongation (Zhou et al, 2020). Interestingly, we found that the E1 ubiquitin-activating enzyme Uba1 was early-oxidized during ageing. Uba1 was recently found to form a disulphide bond with the ubiquitin ligase Rad6, which is a determinant of ubiquitinated ribosomes. This reversible thiol modification between Uba1 and Rad6 inhibited K63-linked ubiquitin modification (Simoes et al, 2022). A possible implication of this mechanism during ageing remains to be discovered.

As cells get older and enter the middle stage of ageing, a group of folding chaperones is increasingly oxidized (cluster D). Here, protein chaperones downstream of the protein synthesis machinery are affected. Chaperones of the Hsp70 family in general, including Ssz1 and Ssb1 identified in this study, were shown previously to act as redox sensors (Zhang et al, 2022). Although often the specific physiological consequences of cysteine residue modification of particular Hsp70s are unknown, their oxidation can change the fate of their targets, thereby amplifying redox signalling to a broader range of proteins achieving a greater antioxidative potential (Zhang et al, 2022). This includes the increased expression of chaperones. Newly synthesized Hsp70 chaperones are not oxidized and can prevent protein aggregation and/or facilitate the degradation of oxidized and possibly damaged proteins (Kalmar & Greensmith, 2009). Moreover, we found that subunits of the TRiC/CCT complex showed increased oxidation on evolutionarily conserved cysteine residues. The TRiC/CCT complex is an ATP-dependent chaperonin responsible for folding of a variety of cellular proteins (Gestaut et al, 2019). Its potential regulation by oxidation is unknown, but because of its high abundance in the cell, it might also act as a cellular redox sensor. Thus, our analysis provides a rich resource of redox-sensitive key proteins during the early phase of yeast ageing, which are known to govern processes of the protein homeostasis network.

Because our study is limited to the mere identification and quantification of oxidative thiol modifications and their dynamics during ageing, for many examples we can only speculate about the role of the shift in the redox state. Thus, follow-up studies will be important to address the question of whether the increase in the oxidized pool of a particular protein acts as a stress response to alleviate detrimental effects by restoring protein homeostasis during ageing or is a driving force of the ageing process.

## Materials and Methods

### Yeast growth conditions

For OxICAT analysis, *S. cerevisiae* strain YPH499 (*MATa*, *ade2-101*, *his3-Δ200*, *leu2-Δ1*, *ura3-52*, *trp1-Δ63*, *lys2-801*) (Sikorski & Hieter, 1989) was grown in three biological replicates at 28°C on minimal synthetic medium (0.67% [wt/vol] yeast nitrogen base, 0.079% [wt/vol] complete supplement mixture amino acid mix, 20 mg/litre [wt/vol] adenine) containing 2% (vol/vol) glucose. Samples were harvested by centrifugation (3,500*g*, 5 min) at the log phase (OD_600_ ∼ 0.45), when they had reached the stationary phase (=Ageing Day 0, OD_600_ ∼ 7) and at days 3, 6, and 9 during the stationary phase. Pellets from 50 ml culture (Log) and 10 ml culture (days 0, 3, 6, 9) were resuspended in 10% TCA and 150 mM NaCl, frozen in liquid nitrogen, and stored at −80°C.

For independent yeast survival analysis, *S. cerevisiae* strains YPH499 and W303-1A (*MATa*, *leu2-3/112*, *ura3-1*, *trp1-1*, *his3-11/15*, *ade2-1*, *can1-100*) (Rothstein, 1983) were grown in six and four biological replicates, respectively, at 28°C on a minimal synthetic medium (0.67% [wt/vol] yeast nitrogen base, 0.079% [wt/vol] complete supplement mixture amino acid mix, 20 mg/litre [wt/vol] adenine) containing 2% (vol/vol) glucose. Six replicates for the YPH499 strain were obtained from two independent experiments, each with three biological replicates. Four replicates for the W303-1A strain were obtained from two independent experiments: the first one with three replicates and the second one with one replicate.

### Yeast survival assay

To determine yeast viability, one OD600 unit from each culture of chronologically aged yeast was collected by centrifugation at 3,000*g* for 5 min at RT. Pellets were resuspended in 1 ml of 1x PBS (137 mM NaCl, 12 mM KPO_4_, and 2.7 mM KCl, pH 7.4). Propidium iodide (cat. no. P4170, 3 μg/ml final concentration; Sigma-Aldrich) was added to the cell suspension and incubated for 15 min at RT while protected from light. Propidium iodide stains only dead cells. A negative control (no staining with propidium iodide) and a positive control were prepared for each sample. For the positive control, yeast cells were heat-killed by incubation at 80°C for 2 min. Samples were diluted 1:10 in 1x PBS and kept on ice and analysed within 1 h after staining. All samples were analysed by flow cytometry (BD FACSCalibur). A total of 30,000 cells per sample were analysed. The number of dead cells was recorded using BD CellQuest Pro software.

### Determination of entry into the stationary phase

To determine the entry into the stationary phase, we employed three methods: (1) measurement of glucose concentration in the medium, (2) measurement of growth rate, and (3) quantification of percentage of budding cells (budding index). For measurement of glucose in the medium, 1 ml of yeast culture was spun at 1,700*g* for 4 min and 1 μl of the supernatant in two technical replicates for three biological replicates per time point was used to measure the glucose concentration by an Accu-Chek Instant meter (Roche). Glucose concentration was measured at six time points: after moving to fresh medium (=0 h, day −2), at the time of collecting the log phase sample (=6 h), at the beginning of the stationary phase (=24 h, day −1), and at days 0 (48 h), 2, and 4 of chronological ageing. For measurement of the growth rate, the optical density (OD) of 600 nm was measured using NanoPhotometer NP80 UV-Vis Spectrophotometer (Implen). The measurement was performed in 1:10 diluted yeast samples for six biological replicates per time point for the YPH499 strain and four biological replicates for W303-1A strain. To determine the proliferation rate, we calculated the budding index (Hubler et al, 1993; Fuge et al, 1994; Allen et al, 2006; Weinberger et al, 2007; Arguello-Miranda et al, 2022). We visually determined the number of cells exhibiting budding under the Opta-Tech microscope with a 40x phase-contrast objective. 0.5 ml of yeast culture was vortexed strongly for 1 min to unclamp unbudded cells. The drop of the culture was moved to the glass slide, and 200 cells from at least three different fields of view per three biological replicates per time point were quantified. As a budded cell, we considered each cell with an attached bud of size smaller than the mother cell. We quantified the budding index (% of budded cells) for time points: 0 h (day −2), 6 h, 24 h (day −1), 48 h (day 0 of chronological ageing), and every second day until the end of the time course (until day 17). It is important to note that yeast chronological ageing is characterized by the constant presence of a small fraction of budding cells that might have been improperly arrested in the S/G2 phase of the cell cycle (Allen et al, 2006; Weinberger et al, 2007). The percentage of cells with a bud remained at the level close to 10% during the entire chronological ageing experiment, similarly as reported before for the stationary phase (Arguello-Miranda et al, 2022).

### ROS measurement

ROS levels in YPH499 were assessed according to the method described previously (Topf et al, 2018) with few modifications. Two OD_600_ were collected, and cells were washed with PBS (137 mM NaCl, 12 mM phosphate, and 2.7 mM KCl, pH 7.4). Yeast cells were resuspended in PBS containing 5 µM dihydroethidium (cat. no. D7008; Sigma-Aldrich) or 10 μM CM-H2DCFDA (cat. no. C68272; Invitrogen). The following control samples were prepared for each condition: PBS only, yeast cells resuspended in PBS without dye, and PBS only with dye. Samples were incubated for 40 min at RT in the dark before measurement. Fluorescence was measured for CM-H2DCFDA at an excitation wavelength of 488 nm and an emission wavelength of 533 nm and for dihydroethidium at an excitation wavelength of 535 nm and an emission wavelength of 635 nm using a plate fluorometer (SpectraMax iD3, Molecular Devises). Fluorescent signals were collected for 2 h in 5-min intervals. Fluorescent units of each sample were corrected for autofluorescence and autoxidation of the dye in PBS. Stock solutions of dihydroethidium and CM-H2DCFDA were kept under argon to minimize their exposure to air. Corrected data are presented as the mean ± SD of three biological replicates. Differences between slope values were tested for significance using ANOVA followed by Tukey’s test.

### OxICAT labelling

Protein extraction from whole cells, differential thiol trapping, tryptic digest, peptide enrichment, and cleavage of the biotin tag were performed exactly as described before (Topf et al, 2018). Briefly, samples were homogenized in 10% TCA using glass beads. Protein concentration was determined by the Bradford assay. 200 μg aliquots were centrifuged (21,000*g*, 15 min, 4°C), washed with 5% ice-cold TCA, and resuspended in a mixture of 160 μl denaturing alkylation buffer (DAB, 6 M urea, 200 mM Tris–HCl, pH 8.5, 10 mM EDTA, and 0.5% SDS) with 2 units of heavy ICAT reagent (ABSciex) dissolved in 40 μl acetonitrile. Samples were incubated for 2 h at 37°C under vigorous shaking. Proteins were precipitated by acetone and resuspended in a mixture of 160 μl DAB with 2 units of light ICAT reagent dissolved in 40 μl acetonitrile. TCEP was added to a final concentration of 1 mM, and samples were incubated for 2 h at 37°C under vigorous shaking followed by acetone precipitation. Protein pellets were digested in 200 μl of 20 mM ammonium bicarbonate (NH_4_HCO_3_) with 12 μg Trypsin (sequencing-grade modified, Promega). After 16-h incubation at 37°C, insoluble proteins were pelleted by centrifugation (21,000*g*, 1 min, RT), resuspended in 50 μl of 60% (vol/vol) methanol and 20 mM NH_4_HCO_3_, and incubated with 1 μg Trypsin at 42°C for 3 h. Supernatants of digests in 20 mM NH_4_HCO_3_ and in 60% (vol/vol) methanol and 20 mM NH_4_HCO_3_ were combined. The clean-up of the digests by strong cation exchange chromatography, enrichment by avidin affinity chromatography, and cleavage of the biotin tag were performed using the ICAT kit (ABSciex) following the manufacturer’s instructions.

### LC-MS analysis

Nano-HPLC-ESI-MS/MS analyses were performed with an Orbitrap Elite mass spectrometer connected to an UltiMate 3000 RSLCnano HPLC system (Thermo Fisher Scientific) exactly as described before for whole-cell OxICAT samples (Topf et al, 2018). Peptide samples were resuspended in 30 μl 0.1% TFA, and each biological replicate was analysed in two technical replicates.

### Mass spectrometry data analysis

Raw files of the LC-MS/MS runs from two technical replicates for each of three biological replicates per time point were jointly processed using MaxQuant v 2.0.2.0 (Cox & Mann, 2008). Peptides corresponding to the *S. cerevisiae* protein database (UniProtKB canonical set for strain ATCC 204508/S288c, Proteome ID UP000002311) were retrieved on 2 May 2022 (6,091 entries) using Andromeda (Cox et al, 2011). ICAT light tag (C_10_H_17_N_3_O_3_, 227.13 Da) and ICAT heavy tag (^13^C_9_CH_17_N_3_O_3_, 236.16 Da) were set as light and heavy labels, respectively, with cysteine residue specificity. Up to five labelled amino acids per peptide were allowed. Trypsin was set for the generation of theoretical peptides, allowing a maximum of two missed cleavages. Oxidation of methionine was included as a variable modification. Labelling ratio estimation was performed using the options “Re-quantify” and “Match between runs” with a retention time window of 0.7 min. The precursor mass tolerance was set to 20 ppm for the “first search” option of Andromeda and to 4.5 ppm for the main search. The minimum peptide length was set to six amino acids. A peptide-spectrum match false discovery rate of 1% was applied using the decoy mode “Revert.”

The MaxQuant file “evidence.txt” was used for subsequent analysis. Entries from potential contaminants, decoy hits, and evidence with no quantified intensity were removed. Intensities for the heavy- or light-labelled peptides of 0, resulting from MaxQuant identifying but not quantifying a peptide, were set to NA. Peptide starting positions (i.e., the sequence index of the first amino acid of the peptide) were extracted from the MaxQuant file “peptides.txt” and used for calculating absolute positions. Cysteine identifications (CysIDs) for all peptides containing a cysteine residue were generated from the protein identifier of the leading razor protein and the absolute position of the cysteine included in the peptide. Peptides with multiple cysteine residues were assigned a CysID including all identified residues. Next, % light intensity was calculated for each evidence by dividing the intensity of light-labelled peptides by the sum of intensities of light- and heavy-labelled peptides. ISO-MSMS evidence with labelling state 0 (i.e., only the light labelling partner was identified), which exhibited % light intensities below 50% after re-quantification, was removed. Likewise, ISO-MSMS evidence with labelling state 1 (i.e., only the heavy labelling partner was identified), which exhibited % light intensities above 50% after re-quantification, was removed. MULTI-MATCH evidence and MULTI-SECPEP evidence were filtered out if each ISO-MSMS evidence identified for the cysteine residue (i.e., miscleaved, methionine oxidation, different *Z*-values) was observed with labelling state 0 and % light intensity < 50% or respectively labelling state 1 and % light intensity > 50%. The resulting table contained 224,469 entries (corresponding to 97.2 % of all identified cysteine-containing evidence with non-zero intensity) and was used to calculate the proportion of reversibly oxidized cysteine residues, which we refer to as % oxidation, for each unique cysteine residue. For each time point and biological replicate, total intensities (i.e., light + heavy) and light intensities respectively of both technical replicates were summed up over all evidence (i.e., modified, miscleaved, different charge states) of a given CysID. The % oxidation was calculated as the sum of light intensity divided by the sum of total intensity (light + heavy) multiplied by 100 (for a detailed description, see Topf et al [2018]). Unfiltered intensity values of all biological replicates are reported in Table S1.

For downstream analysis, all raw values corresponding to day 3, biological replicate 3, were removed as this replicate showed disproportionately higher missing values (27% for light-labelled and 33 % for heavy-labelled CysID evidence) compared with the other replicates (<5% for light- and heavy-labelled CysID evidence, respectively). Furthermore, CysIDs with missing heavy or light intensities in more than one biological replicate were removed. In total, 65.5% of all peptides were removed after filtering. 3,178 peptides of a total of 9,213 remained for further downstream analysis. We refer to this dataset as the “yeast OxiAge” dataset (Table S2).

### Missing value imputation

Missing intensity values for light or heavy ICAT-labelled peptides resulting from unidentified or non-quantified peptides were imputed using a data-driven imputation strategy (Egert et al, 2021). The filtered data frame was used as input for the DIMA protocol implemented in R (version 4.1.0). Briefly, DIMA uses a subset of the data frame with no missing values as a template to generate a benchmarking dataset with the same distribution of missing values as the original dataset. Various imputation algorithms are tested on the benchmark dataset and evaluated based on the known true values. The best-scoring algorithm is used for the imputation of the initial dataset. For the OxICAT dataset, the impSeqRob algorithm from the R package rrcovNA (Verboven et al, 2007; Branden & Verboven, 2009) was consistently best-performing. Using this imputation method, a total of 2,345 intensity values for light and heavy ICAT-labelled peptides were generated (2.64% of all intensity values), corresponding to 1,229 values of % oxidation (2.76% of all oxidation values). A dataset with imputed values was used to generate PCA of components 1 and 2 for each replicate and each time point and volcano plots of statistically significant changes between each pair of time points.

### Peptide clustering

All downstream analyses of the filtered mass spectrometry dataset were performed in Python, version 3.9, unless stated otherwise. For the clustering analysis, peptides of the “yeast OxiAge” dataset with not imputed values were used for downstream analysis. Only peptides with quantified % oxidation in at least two biological replicates at each time point were included in the clustering analysis. Peptides were clustered into 10 groups based on the mean change in % oxidation over time in a two-step process. Firstly, outliers that could impact the clustering procedure were determined using the DBSCAN algorithm implemented in the Python package sklearn (https://github.com/scikit-learn/scikit-learn, Pedregosa et al, 2011) with a maximum distance between neighbouring samples set to 22 and the total weight set to 13. Six outliers were identified and removed from the clustering dataset. Secondly, peptides after the removal of the outliers were grouped using StandardScaler for segmentation followed by Gaussian mixture (GM) for clustering, both algorithms implemented in the sklearn package. Peptides were grouped into four initial clusters, as determined by the Elbow method, with random seed set to 8,000, convergence threshold set to 1, and covariance type set to *diag* (diagonal covariance matrix per component). Clusters were reviewed manually, and standard deviations between clusters were compared. Clusters exhibiting non-homogeneous trends were re-clustered using the same parameters and the random seed set to 10,000. The resulting 10 clusters were manually labelled A–J based on the trend of an early increase in % oxidation. The six outliers removed from the dataset before clustering were manually added to the cluster containing the highest variation between peptides with no obvious trends (cluster I). The method of clustering, the number of clusters used, and the exact parameters’ values were decided by the try-and-check method.

### Annotation analysis

Gene Ontology (GO) annotations for cellular components (CC) of yeast proteome were retrieved on 14.03.2022 from UniProtKB for strain ATCC 204508/S288c, Proteome ID UP000002311. For cellular localization, GO annotations were consolidated into nine main annotations according to parent–child terms, using as the highest degree parent terms corresponding to Cytoplasm (GO:0005737), Nucleus (GO:0005634), Endoplasmic reticulum (ER, GO:0005783), Golgi apparatus (GO:0005794), Mitochondrion (GO:0005739), Vacuole (GO:0005773), Peroxisome (GO:0005777; GO:0019818), Lysosome (GO:0005764), and Ribosome (GO:0005840; GO:0033279). An additional group named “Other” contained all proteins with other localizations specified that were not considered in this study, such as the extracellular region. Proteins annotated with both terms “Cytoplasm” and “Nucleus” were grouped into a separate term “Nucleus/Cytoplasm.” Proteins associated with cytosolic and mitochondrial ribosomes were grouped into the term “Ribosome.” Proteins associated with Cytoplasm and a second additional organelle except for Nucleus were considered annotated to the latter group; that is, a protein assigned to Cytoplasm and ER was grouped as ER-localized. Proteins assigned to multiple organelles were grouped under the term “Multiple localization.” Proteins without known GO annotation were described as “Unknown.”

To identify proteins involved in proteostasis, we used GO Slim Yeast annotations retrieved from QuickGO (Binns et al, 2009) on 27.10.2022. We grouped proteins according to one of three groups of biological processes (BP) regulating proteostasis: cytoplasmic translation, protein degradation, or protein folding. For global translation, we searched for the words “translation,” “translational” etc. (exception: mitochondrial-specific translation). For protein degradation, we searched for the words “ubiquitin,” “ubiquitination,” “polyubiquitination,” “proteasome,” “proteasomal,” “protein conjugation,” and “proteolysis.” For protein folding, we looked for the words “folding” and “refolding.” Results were manually reviewed for uncertain proteins. Proteins within each cluster (A–J) were grouped based on one of the three proteostasis groups.

Information about disulphide bonds, protein domains, length, sequence, isomers, structures, interactions, etc., was retrieved from UniProtKB on dates 14.11.2022–03.02.2023.

### GO enrichment analysis

Analysis of the overrepresentation of GO terms was performed using the TopGo package in R (version 4.1.0). For each cluster, a custom analysis was performed using as an input species yeast *S. cerevisiae* and as a background cysteine-containing proteins according to amino acid sequences provided from the yeast proteome (yeast proteome retrieved on 14.03.2022 from UniProtKB for strain ATCC 204508/S288c, Proteome ID UP000002311). We identified 5,478 canonical proteins as containing at least one cysteine residue within the provided amino acid sequence. GO Slim annotations were retrieved from QuickGO (Binns et al, 2009) on 27.10.2022. GO biological process (BP) and GO molecular function (MF) were searched for enrichment. GO terms with at least 20 genes per term were used, and the algorithm *weight01* with Fisher’s statistics was used to calculate the *P*-value for each term. The top 200 terms were selected, and *P*-value correction was performed on GO terms with fold enrichment > 1.5 and *P*-value < 0.05. Benjamini–Hochberg’s (BH) procedure was used as a correction for multiple testing for the identification of significant enrichment. In addition, Bonferroni and Hochberg’s corrections were performed. The fold enrichment was calculated as the number of significant genes found in a GO term divided by the number of expected genes taken from the submitted background. Fold enrichment is a measure that indicates how drastically proteins belonging to a certain pathway are overrepresented independently of the pathway size. To simplify and summarize the high number of enriched processes, the BP terms were grouped using the online tool Revigo (Supek et al, 2011), with the GO database updated on 03.2022 and UniProt-to-GO mapping from EBI GOA updated on 04.2022. *S. cerevisiae* was used as a species, the Lin method was used as a semantic similarity measure, and the *C*-value was set to 0.5. For visualization purposes, a representative GO term for each Revigo group was chosen based on the lowest *P*-value.

### Identification of zinc-binding cysteine residues

To determine zinc-binding proteins, the dataset established by Wang et al (2018) was used. All oxidized cysteine residues per each quantified protein from the “yeast OxiAge” dataset were mapped into known or potential zinc-binding proteins (582 proteins) and zinc-dependent proteins identified by mass spectrometry analysis of protein abundance in zinc-depleted cells (over 2,500 proteins identified).

### Protein association network

Cytoscape 3.9.1. (Shannon et al, 2003) with plug-in StringApp 1.7.1. (Doncheva et al, 2019) was used to generate a functional protein association network. The minimum required interaction score was set to confidence 0.6 unless specified differently. A full STRING network was applied with the meaning of network edges set to “confidence,” meaning that the thickness of lines indicates the strength of evidence in the literature. For the display, the structure previews were disabled. A compound spring embedder layout was used for visualization. Proteins were coloured based on the biological process of proteostasis: translation in blue, folding in green, and/or degradation in red. Proteins that belong to more than one group had nodes coloured with more than one colour.

### Motif analysis

Analysis of motifs was performed by extracting motifs centred ±6 amino acids around each quantified cysteine residue in the “yeast OxiAge” dataset. The analysis and visualization of motifs with a total length of 13 amino acids were performed using the freely available online tool pLogo (O’Shea et al, 2013). Overrepresented and underrepresented motifs were analysed for each A–J cluster separately. Cysteine residues detected in more than one peptide were used only once. Sequences with cysteine residues closer to the START or STOP codons than 6 amino acids were removed from the analysis. As background, sequences in the FASTA format of yeast proteins containing cysteine residues (obtained from yeast proteome in UniProtKB as described before) were used. Redundancy was removed from the background and the foreground. Forward sequences were not removed from the background. Amino acids with *P*-values < 0.05 were considered significant. Colours were assigned to each amino acid: non-polar amino acids are in black except G and A coloured in green; sulphur-containing methionine (M) is in black; polar uncharged are in orange; aromatic are in grey; positively charged are in red; negatively charged are in blue; and sulphur-containing cysteine (C) is in yellow. Log-odds binomial probabilities were shown, and frequencies of each overrepresented amino acid were extracted.

### Ortholog analysis

To compare data between different species, the ortholog file was downloaded from the Alliance of Genome Resources on 16.05.2022 (Alliance Database Version 5.2.0; species: *Homo sapiens*, *Rattus norvegicus*, *M. musculus*, *Danio rerio*, *D. melanogaster*, *C. elegans*, *S. cerevisiae*). Canonical forms of proteins quantified in the yeast dataset were searched for orthologs in other species based on identification numbers from a specific organismal database: Saccharomyces Genome Database, Mouse Genome Informatics, WormBase: Nematode Information Resource (WB), and FlyBase (FB). The data were compared between different species of datasets: “yeast OxiAge” (this study), yeast strain DBY746 (Brandes et al, 2013), nematode (Knoefler et al, 2012), fruit fly (Menger et al, 2015), and mice (Xiao et al, 2020). The four latter datasets were filtered before the ortholog analysis. For yeast strain DBY746, data were retrieved from the experiment performed in standard conditions for chronological ageing (2% glucose). Values for % oxidation at the logarithmic phase (Log), and days 1, 2, 3, and 4 were obtained. Peptides with unidentified or unquantified average % oxidation values in any of the given time points were removed from the analysis. UniProt IDs were manually checked and updated to the current state of the database on day 24.10.2022. Obsolete terms were removed. In total, 287 peptides were retrieved. For nematode *C. elegans* strain N2 “Bristol,” data were retrieved from the experiment performed in WT animals from the larva stage L4 and worms at days 2, 8, and 15 of adulthood. Filtering of the peptides was performed as described before for yeast strain DBY476. UniProt IDs were manually checked and updated, and obsolete terms were removed, as described before. In total, 108 peptides were retrieved. For fruit flies, data from WT days 7, 28, and 56 of adulthood were retrieved for control strains, ageing. We considered only peptides with identified values for three time points. 252 peptides were retrieved. For male mice, *M. musculus*, strain C57BL/6, data on young and old mice were obtained from the analysis performed on 10 different tissues in animals aged 16 and 80 wk. Filtering of the peptides was performed as described before. UniProt IDs were manually checked and updated, and obsolete terms were removed, as described before, for each time point and tissue. In total, 60,841 peptides for different tissues were retrieved, which corresponds to 21,850 unique peptides in all tissues. Evolutionarily conserved proteins found oxidized in different species were labelled as “common proteins.” For comparison of evolutionarily conserved cysteine residues, sequence alignment was performed (see below) between yeast proteins involved in proteostasis and corresponding proteins in worms, fruit flies, and mice identified by ortholog search. Positions of the aligned cysteine residues were matched to the positions of the residues of oxidized cysteines within different datasets. Conserved cysteine residues found oxidized in different species were labelled as “common cysteines.” To visualize the overlap between species, all oxidized proteins in the worm, fruit fly, and mouse datasets were labelled according to the yeast ortholog UniProt ID, then mouse and worm, unless no orthologs were found.

### Sequence alignment

Amino acid sequence alignment was performed using Biotite framework version 0.35.0 (Kunzmann & Hamacher, 2018) implemented in Python, Biopython version 1.80 (Cock et al, 2009). Clustal Omega (Sievers & Higgins, 2021) was performed for multiple sequence alignment with a Python package clustalo.ClustalOmegaApp using software Clustal Omega, version 1.2.2. For pairwise alignment, packages align_optimal and SubstitutionMatrix.std_protein_matrix from Biotite.align were used.

### Database and web application

The web application (http://oxiage.ibb.waw.pl) is currently hosted on the Institute of Biochemistry and Biophysics Xen 6.2 virtualization on the Ubuntu 22.04 LTS operating system, Apache 2 version 2.4 wsgi modem. It is developed using Python Interpreter version 3.10 in the Dash framework version 2.7.1 (https://github.com/plotly/dash). For display and user interaction, JavaScript is used. Cascading Style Sheets is used for styling website components. Graphs and charts are displayed using plotly.js (https://github.com/plotly/plotly.js/). For responsive layout, Dash bootstrap components version 1.3.0 (https://github.com/facultyai/dash-bootstrap-components), Dash mantine components version 0.11.1 (https://github.com/snehilvj/dash-mantine-components), and Dash HTML components version 2.0.0 (https://github.com/plotly/dash-html-components) are used. To visualize data frames and tables, pandas package version 1.5.2 (https://github.com/pandas-dev/pandas) and Dash table components version 5.0.0 (https://github.com/plotly/dash-table) are used. Sequence alignment is performed using Biotite.align from Biotite framework version 0.35.0 (Kunzmann & Hamacher, 2018). The database containing static resources (i.e., protein name, protein sequence, detected cysteine sites) is developed using a structural query language (SQL) and a relational database management system MySQL 8.0. Connection to MySQL database is established using MySQL Connector/Python version 8.0.31 (https://github.com/mysql/mysql-connector-python). For a detailed description of the use and features of the database and the web application, please visit the help page of the website.

### Statistical analysis

A number of sample sizes, replicates, and statistical tests were chosen according to published data with comparable methodology. No statistical method was used to predetermine the sample size. ANOVA followed by Tukey’s test was used to compare between slope values of the fluorescent signal of ROS measurement with dihydroethidium and CM-H2DCFDA. For statistical analysis of oxidized peptides in filtered “yeast OxiAge” dataset, imputed values were used. Statistical significance between each time point group was calculated using a two-sided Welch *t* test for an unequal variance for imputed data. Volcano plots were used to visualize the data and present −log_10_ of *P*-value on the *y*-axis and the difference in mean % oxidation between each pair. PCA for components 1 and 2 was generated using MaxQuant/Perseus software version 2.0.9.0 (Tyanova et al, 2016). Pearson’s correlation coefficient between all replicates of imputed and non-imputed data was calculated using the Python package sklearn (https://github.com/scikit-learn/scikit-learn, Pedregosa et al, 2011). Statistical analysis of differences in % oxidation of peptides between time points per each cluster A–J was performed using the Kruskal–Wallis test with Dunn’s post hoc method. The Bonferroni correction was applied for multiple testing, and *P*-values were considered significant if <0.005. For GO enrichment analysis, Fisher’s exact test was used as described before to identify enriched GO terms. The Benjamini–Hochberg (BH) procedure was used to correct for multiple testing and identify significantly enriched GO terms. A significant difference in the overrepresentation of motifs using pLogo was calculated as described in O’Shea et al (2013). The statistical significance threshold was calculated using the Bonferroni correction. The *y*-axis denotes the log-odds binomial probability. Residues are stacked from most to least significant.

## Data Availability

The mass spectrometry proteomics data have been deposited to the ProteomeXchange Consortium (Deutsch et al, 2022) via the PRIDE (Perez-Riverol et al, 2022) partner repository with the dataset identifier PXD042047. The web source code is available via GitHub: https://github.com/katarzynajonak/oxiage. The web server is available at: http://oxiage.ibb.waw.pl. The website is free and open to all users. All other data needed to evaluate the conclusions in the article are present in the article and/or the Supplementary Materials.

## Supplementary Material

Reviewer comments

## References

[bib1] Agarwal S, Sohal RS (1996) Relationship between susceptibility to protein oxidation, aging, and maximum life span potential of different species. Exp Gerontol 31: 365–372. 10.1016/0531-5565(95)02039-x 9415119

[bib2] Allen C, Buttner S, Aragon AD, Thomas JA, Meirelles O, Jaetao JE, Benn D, Ruby SW, Veenhuis M, Madeo F, (2006) Isolation of quiescent and nonquiescent cells from yeast stationary-phase cultures. J Cell Biol 174: 89–100. 10.1083/jcb.200604072 16818721 PMC2064167

[bib3] Anisimova AS, Alexandrov AI, Makarova NE, Gladyshev VN, Dmitriev SE (2018) Protein synthesis and quality control in aging. Aging (Albany NY) 10: 4269–4288. 10.18632/aging.101721 30562164 PMC6326689

[bib4] Antelmann H, Helmann JD (2011) Thiol-based redox switches and gene regulation. Antioxid Redox Signal 14: 1049–1063. 10.1089/ars.2010.3400 20626317 PMC3113447

[bib5] Araki K, Kusano H, Sasaki N, Tanaka R, Hatta T, Fukui K, Natsume T (2016) Redox sensitivities of global cellular cysteine residues under reductive and oxidative stress. J Proteome Res 15: 2548–2559. 10.1021/acs.jproteome.6b00087 27350002

[bib6] Arguello-Miranda O, Marchand AJ, Kennedy T, Russo MAX, Noh J (2022) Cell cycle-independent integration of stress signals by Xbp1 promotes Non-G1/G0 quiescence entry. J Cell Biol 221: e202103171. 10.1083/jcb.202103171 34694336 PMC8548912

[bib7] Begas P, Liedgens L, Moseler A, Meyer AJ, Deponte M (2017) Glutaredoxin catalysis requires two distinct glutathione interaction sites. Nat Commun 8: 14835. 10.1038/ncomms14835 28374771 PMC5382279

[bib8] Binns D, Dimmer E, Huntley R, Barrell D, O’Donovan C, Apweiler R (2009) QuickGO: A web-based tool for gene ontology searching. Bioinformatics 25: 3045–3046. 10.1093/bioinformatics/btp536 19744993 PMC2773257

[bib9] Bonawitz ND, Chatenay-Lapointe M, Pan Y, Shadel GS (2007) Reduced TOR signaling extends chronological life span via increased respiration and upregulation of mitochondrial gene expression. Cell Metab 5: 265–277. 10.1016/j.cmet.2007.02.009 17403371 PMC3460550

[bib10] Branden KV, Verboven S (2009) Robust data imputation. Comput Biol Chem 33: 7–13. 10.1016/j.compbiolchem.2008.07.019 18771957

[bib11] Brandes N, Reichmann D, Tienson H, Leichert LI, Jakob U (2011) Using quantitative redox proteomics to dissect the yeast redoxome. J Biol Chem 286: 41893–41903. 10.1074/jbc.M111.296236 21976664 PMC3308895

[bib12] Brandes N, Tienson H, Lindemann A, Vitvitsky V, Reichmann D, Banerjee R, Jakob U (2013) Time line of redox events in aging postmitotic cells. Elife 2: e00306. 10.7554/eLife.00306 23390587 PMC3564446

[bib13] Burri L, Vascotto K, Fredersdorf S, Tiedt R, Hall MN, Lithgow T (2004) Zim17, a novel zinc finger protein essential for protein import into mitochondria. J Biol Chem 279: 50243–50249. 10.1074/jbc.M409194200 15383543

[bib14] Calabrese G, Morgan B, Riemer J (2017) Mitochondrial glutathione: Regulation and functions. Antioxid Redox Signal 27: 1162–1177. 10.1089/ars.2017.7121 28558477

[bib15] Chacinska A, Pfannschmidt S, Wiedemann N, Kozjak V, Sanjuan Szklarz LK, Schulze-Specking A, Truscott KN, Guiard B, Meisinger C, Pfanner N (2004) Essential role of Mia40 in import and assembly of mitochondrial intermembrane space proteins. EMBO J 23: 3735–3746. 10.1038/sj.emboj.7600389 15359280 PMC522791

[bib16] Chae HZ, Chung SJ, Rhee SG (1994a) Thioredoxin-dependent peroxide reductase from yeast. J Biol Chem 269: 27670–27678. 10.1016/s0021-9258(18)47038-x 7961686

[bib17] Chae HZ, Uhm TB, Rhee SG (1994b) Dimerization of thiol-specific antioxidant and the essential role of cysteine 47. Proc Natl Acad Sci U S A 91: 7022–7026. 10.1073/pnas.91.15.7022 8041739 PMC44330

[bib18] Chiocchetti A, Zhou J, Zhu H, Karl T, Haubenreisser O, Rinnerthaler M, Heeren G, Oender K, Bauer J, Hintner H, (2007) Ribosomal proteins Rpl10 and Rps6 are potent regulators of yeast replicative life span. Exp Gerontol 42: 275–286. 10.1016/j.exger.2006.11.002 17174052

[bib19] Cock PJA, Antao T, Chang JT, Chapman BA, Cox CJ, Dalke A, Friedberg I, Hamelryck T, Kauff F, Wilczynski B, (2009) Biopython: Freely available Python tools for computational molecular biology and bioinformatics. Bioinformatics 25: 1422–1423. 10.1093/bioinformatics/btp163 19304878 PMC2682512

[bib20] Cooke MS, Evans MD, Dizdaroglu M, Lunec J (2003) Oxidative DNA damage: Mechanisms, mutation, and disease. FASEB J 17: 1195–1214. 10.1096/fj.02-0752rev 12832285

[bib21] Cox J, Mann M (2008) MaxQuant enables high peptide identification rates, individualized p.p.b.-range mass accuracies and proteome-wide protein quantification. Nat Biotechnol 26: 1367–1372. 10.1038/nbt.1511 19029910

[bib22] Cox J, Neuhauser N, Michalski A, Scheltema RA, Olsen JV, Mann M (2011) Andromeda: A peptide search engine integrated into the MaxQuant environment. J Proteome Res 10: 1794–1805. 10.1021/pr101065j 21254760

[bib23] Crosas B, Hanna J, Kirkpatrick DS, Zhang DP, Tone Y, Hathaway NA, Buecker C, Leggett DS, Schmidt M, King RW, (2006) Ubiquitin chains are remodeled at the proteasome by opposing ubiquitin ligase and deubiquitinating activities. Cell 127: 1401–1413. 10.1016/j.cell.2006.09.051 17190603

[bib24] Culotta VC, Klomp LW, Strain J, Casareno RL, Krems B, Gitlin JD (1997) The copper chaperone for superoxide dismutase. J Biol Chem 272: 23469–23472. 10.1074/jbc.272.38.23469 9295278

[bib25] Curran SP, Ruvkun G (2007) Lifespan regulation by evolutionarily conserved genes essential for viability. PLoS Genet 3: e56. 10.1371/journal.pgen.0030056 17411345 PMC1847696

[bib26] Czachor J, Milek M, Galiniak S, Stepien K, Dzugan M, Molon M (2020) Coffee extends yeast chronological lifespan through antioxidant properties. Int J Mol Sci 21: 9510. 10.3390/ijms21249510 33327536 PMC7765085

[bib27] D’Silva PD, Schilke B, Walter W, Andrew A, Craig EA (2003) J protein cochaperone of the mitochondrial inner membrane required for protein import into the mitochondrial matrix. Proc Natl Acad Sci U S A 100: 13839–13844. 10.1073/pnas.1936150100 14605210 PMC283508

[bib28] David DC (2012) Aging and the aggregating proteome. Front Genet 3: 247. 10.3389/fgene.2012.00247 23181070 PMC3501694

[bib29] Deutsch EW, Bandeira N, Perez-Riverol Y, Sharma V, Carver JJ, Mendoza L, Kundu DJ, Wang SB, Bandla C, Kamatchinathan S, (2022) The ProteomeXchange consortium at 10 years: 2023 update. Nucleic Acids Res 51: D1539–D1548. 10.1093/nar/gkac1040 PMC9825490 36370099

[bib30] Doncheva NT, Morris JH, Gorodkin J, Jensen LJ (2019) Cytoscape StringApp: Network analysis and visualization of proteomics data. J Proteome Res 18: 623–632. 10.1021/acs.jproteome.8b00702 30450911 PMC6800166

[bib31] Dougherty SE, Maduka AO, Inada T, Silva GM (2020) Expanding role of ubiquitin in translational control. Int J Mol Sci 21: 1151. 10.3390/ijms21031151 32050486 PMC7037965

[bib32] Duncan K, Umen JG, Guthrie C (2000) A putative ubiquitin ligase required for efficient mRNA export differentially affects hnRNP transport. Curr Biol 10: 687–696. 10.1016/s0960-9822(00)00527-3 10873801

[bib33] Egert J, Brombacher E, Warscheid B, Kreutz C (2021) DIMA: Data-driven selection of an imputation algorithm. J Proteome Res 20: 3489–3496. 10.1021/acs.jproteome.1c00119 34062065

[bib34] Eleutherio ECA, Silva Magalhães RS, de Araújo Brasil A, Monteiro Neto JR, de Holanda Paranhos L (2021) SOD1, more than just an antioxidant. Arch Biochem Biophys 697: 108701. 10.1016/j.abb.2020.108701 33259795

[bib35] Fabrizio P, Longo VD (2003) The chronological life span of Saccharomyces cerevisiae. Aging Cell 2: 73–81. 10.1046/j.1474-9728.2003.00033.x 12882320

[bib36] Finelli MJ (2020) Redox post-translational modifications of protein thiols in brain aging and neurodegenerative conditions-focus on S-nitrosation. Front Aging Neurosci 12: 254. 10.3389/fnagi.2020.00254 33088270 PMC7497228

[bib37] Fuge EK, Braun EL, Werner-Washburne M (1994) Protein synthesis in long-term stationary-phase cultures of Saccharomyces cerevisiae. J Bacteriol 176: 5802–5813. 10.1128/jb.176.18.5802-5813.1994 8083172 PMC196785

[bib38] Furukawa K, Mizushima N, Noda T, Ohsumi Y (2000) A protein conjugation system in yeast with homology to biosynthetic enzyme reaction of prokaryotes. J Biol Chem 275: 7462–7465. 10.1074/jbc.275.11.7462 10713047

[bib39] Gestaut D, Roh SH, Ma B, Pintilie G, Joachimiak LA, Leitner A, Walzthoeni T, Aebersold R, Chiu W, Frydman J (2019) The chaperonin TRiC/CCT associates with prefoldin through a conserved electrostatic interface essential for cellular proteostasis. Cell 177: 751–765.e15. 10.1016/j.cell.2019.03.012 30955883 PMC6629582

[bib40] Ghosh A, Trivedi PP, Timbalia SA, Griffin AT, Rahn JJ, Chan SSL, Gohil VM (2014) Copper supplementation restores cytochrome c oxidase assembly defect in a mitochondrial disease model of COA6 deficiency. Hum Mol Genet 23: 3596–3606. 10.1093/hmg/ddu069 24549041 PMC4049311

[bib41] Gladyshev VN, Kritchevsky SB, Clarke SG, Cuervo AM, Fiehn O, de Magalhaes JP, Mau T, Maes M, Moritz RL, Niedernhofer LJ, (2021) Molecular damage in aging. Nat Aging 1: 1096–1106. 10.1038/s43587-021-00150-3 36846190 PMC9957516

[bib42] Glerum DM, Shtanko A, Tzagoloff A (1996) Characterization of COX17, a yeast gene involved in copper metabolism and assembly of cytochrome oxidase. J Biol Chem 271: 14504–14509. 10.1074/jbc.271.24.14504 8662933

[bib43] Go YM, Jones DP (2008) Redox compartmentalization in eukaryotic cells. Biochim Biophys Acta 1780: 1273–1290. 10.1016/j.bbagen.2008.01.011 18267127 PMC2601570

[bib44] Go YM, Duong DM, Peng J, Jones DP (2011) Protein cysteines Map to functional networks according to steady-state level of oxidation. J Proteomics Bioinform 4: 196–209. 10.4172/jpb.1000190 22605892 PMC3352318

[bib45] Gould NS, Evans P, Martinez-Acedo P, Marino SM, Gladyshev VN, Carroll KS, Ischiropoulos H (2015) Site-specific proteomic mapping identifies selectively modified regulatory cysteine residues in functionally distinct protein networks. Chem Biol 22: 965–975. 10.1016/j.chembiol.2015.06.010 26165157 PMC4515171

[bib46] Graille M, Quevillon-Cheruel S, Leulliot N, Zhou CZ, Li de la Sierra Gallay I, Jacquamet L, Ferrer JL, Liger D, Poupon A, Janin J, (2004) Crystal structure of the YDR533c S. cerevisiae protein, a class II member of the Hsp31 family. Structure 12: 839–847. 10.1016/j.str.2004.02.030 15130476

[bib47] Groitl B, Jakob U (2014) Thiol-based redox switches. Biochim Biophys Acta 1844: 1335–1343. 10.1016/j.bbapap.2014.03.007 24657586 PMC4059413

[bib48] Hansen M, Taubert S, Crawford D, Libina N, Lee SJ, Kenyon C (2007) Lifespan extension by conditions that inhibit translation in Caenorhabditis elegans. Aging Cell 6: 95–110. 10.1111/j.1474-9726.2006.00267.x 17266679

[bib49] Hartl FU (2017) Protein misfolding diseases. Annu Rev Biochem 86: 21–26. 10.1146/annurev-biochem-061516-044518 28441058

[bib50] Herker E, Jungwirth H, Lehmann KA, Maldener C, Frohlich KU, Wissing S, Buttner S, Fehr M, Sigrist S, Madeo F (2004) Chronological aging leads to apoptosis in yeast. J Cell Biol 164: 501–507. 10.1083/jcb.200310014 14970189 PMC2171996

[bib51] Herman PK (2002) Stationary phase in yeast. Curr Opin Microbiol 5: 602–607. 10.1016/s1369-5274(02)00377-6 12457705

[bib52] Hipp MS, Kasturi P, Hartl FU (2019) The proteostasis network and its decline in ageing. Nat Rev Mol Cell Biol 20: 421–435. 10.1038/s41580-019-0101-y 30733602

[bib53] Hohn A, Konig J, Grune T (2013) Protein oxidation in aging and the removal of oxidized proteins. J Proteomics 92: 132–159. 10.1016/j.jprot.2013.01.004 23333925

[bib54] Hohn A, Weber D, Jung T, Ott C, Hugo M, Kochlik B, Kehm R, Konig J, Grune T, Castro JP (2017) Happily (n)ever after: Aging in the context of oxidative stress, proteostasis loss and cellular senescence. Redox Biol 11: 482–501. 10.1016/j.redox.2016.12.001 28086196 PMC5228102

[bib55] Hubler L, Bradshaw-Rouse J, Heideman W (1993) Connections between the Ras-cyclic AMP pathway and G1 cyclin expression in the budding yeast Saccharomyces cerevisiae. Mol Cell Biol 13: 6274–6282. 10.1128/mcb.13.10.6274-6282.1993 8413227 PMC364686

[bib56] Ikeuchi K, Tesina P, Matsuo Y, Sugiyama T, Cheng J, Saeki Y, Tanaka K, Becker T, Beckmann R, Inada T (2019) Collided ribosomes form a unique structural interface to induce Hel2-driven quality control pathways. EMBO J 38: e100276. 10.15252/embj.2018100276 30609991 PMC6396155

[bib57] Jahngen-Hodge J, Obin MS, Gong X, Shang F, Nowell TR Jr., Gong J, Abasi H, Blumberg J, Taylor A (1997) Regulation of ubiquitin-conjugating enzymes by glutathione following oxidative stress. J Biol Chem 272: 28218–28226. 10.1074/jbc.272.45.28218 9353272

[bib58] Jumper J, Evans R, Pritzel A, Green T, Figurnov M, Ronneberger O, Tunyasuvunakool K, Bates R, Zidek A, Potapenko A, (2021) Highly accurate protein structure prediction with AlphaFold. Nature 596: 583–589. 10.1038/s41586-021-03819-2 34265844 PMC8371605

[bib59] Kalmar B, Greensmith L (2009) Induction of heat shock proteins for protection against oxidative stress. Adv Drug Deliv Rev 61: 310–318. 10.1016/j.addr.2009.02.003 19248813

[bib60] Kanamori E, Igarashi S, Osawa M, Fukunishi Y, Shimada I, Nakamura H (2011) Structure determination of a protein assembly by amino acid selective cross-saturation. Proteins 79: 179–190. 10.1002/prot.22871 20954264

[bib61] Kapadia BB, Gartenhaus RB (2019) DUBbing down translation: The functional interaction of deubiquitinases with the translational machinery. Mol Cancer Ther 18: 1475–1483. 10.1158/1535-7163.MCT-19-0307 31481479 PMC6727985

[bib62] Katiyar S, Suzuki T, Balgobin BJ, Lennarz WJ (2002) Site-directed mutagenesis study of yeast peptide:N-glycanase. Insight into the reaction mechanism of deglycosylation. J Biol Chem 277: 12953–12959. 10.1074/jbc.M111383200 11812789

[bib63] Kaushik S, Cuervo AM (2015) Proteostasis and aging. Nat Med 21: 1406–1415. 10.1038/nm.4001 26646497

[bib64] Knoefler D, Thamsen M, Koniczek M, Niemuth NJ, Diederich AK, Jakob U (2012) Quantitative in vivo redox sensors uncover oxidative stress as an early event in life. Mol Cell 47: 767–776. 10.1016/j.molcel.2012.06.016 22819323 PMC3444654

[bib65] Kostetsky P, Vladimirova R (1990) Identification of significant conservative and variable regions in homologous protein sequences. Biochimie 72: 295–297. 10.1016/0300-9084(90)90087-w 2166594

[bib66] Krisko A, Radman M (2019) Protein damage, ageing and age-related diseases. Open Biol 9: 180249. 10.1098/rsob.180249 30914006 PMC6451363

[bib67] Kumsta C, Thamsen M, Jakob U (2011) Effects of oxidative stress on behavior, physiology, and the redox thiol proteome of Caenorhabditis elegans. Antioxid Redox Signal 14: 1023–1037. 10.1089/ars.2010.3203 20649472 PMC3052275

[bib68] Kunzmann P, Hamacher K (2018) Biotite: A unifying open source computational biology framework in Python. Bmc Bioinformatics 19: 346. 10.1186/s12859-018-2367-z 30285630 PMC6167853

[bib69] Lamb AL, Torres AS, O’Halloran TV, Rosenzweig AC (2000) Heterodimer formation between superoxide dismutase and its copper chaperone. Biochemistry 39: 14720–14727. 10.1021/bi002207a 11101286

[bib70] Leichert LI, Gehrke F, Gudiseva HV, Blackwell T, Ilbert M, Walker AK, Strahler JR, Andrews PC, Jakob U (2008) Quantifying changes in the thiol redox proteome upon oxidative stress in vivo. Proc Natl Acad Sci U S A 105: 8197–8202. 10.1073/pnas.0707723105 18287020 PMC2448814

[bib71] Leidel S, Pedrioli PG, Bucher T, Brost R, Costanzo M, Schmidt A, Aebersold R, Boone C, Hofmann K, Peter M (2009) Ubiquitin-related modifier Urm1 acts as a sulphur carrier in thiolation of eukaryotic transfer RNA. Nature 458: 228–232. 10.1038/nature07643 19145231

[bib72] Li X, Snyder MP (2016) Yeast longevity promoted by reversing aging-associated decline in heavy isotope content. NPJ Aging Mech Dis 2: 16004. 10.1038/npjamd.2016.4 28721263 PMC5515009

[bib73] Ling JQ, Soll D (2010) Severe oxidative stress induces protein mistranslation through impairment of an aminoacyl-tRNA synthetase editing site. Proc Natl Acad Sci U S A 107: 4028–4033. 10.1073/pnas.1000315107 20160114 PMC2840151

[bib74] Loewith R, Jacinto E, Wullschleger S, Lorberg A, Crespo JL, Bonenfant D, Oppliger W, Jenoe P, Hall MN (2002) Two TOR complexes, only one of which is rapamycin sensitive, have distinct roles in cell growth control. Mol Cell 10: 457–468. 10.1016/s1097-2765(02)00636-6 12408816

[bib75] Longo VD, Shadel GS, Kaeberlein M, Kennedy B (2012) Replicative and chronological aging in Saccharomyces cerevisiae. Cell Metab 16: 18–31. 10.1016/j.cmet.2012.06.002 22768836 PMC3392685

[bib76] Lopez-Otin C, Blasco MA, Partridge L, Serrano M, Kroemer G (2023) Hallmarks of aging: An expanding universe. Cell 186: 243–278. 10.1016/j.cell.2022.11.001 36599349

[bib77] Luikenhuis S, Perrone G, Dawes IW, Grant CM (1998) The yeast Saccharomyces cerevisiae contains two glutaredoxin genes that are required for protection against reactive oxygen species. Mol Biol Cell 9: 1081–1091. 10.1091/mbc.9.5.1081 9571241 PMC25331

[bib78] MacLean M, Harris N, Piper PW (2001) Chronological lifespan of stationary phase yeast cells; a model for investigating the factors that might influence the ageing of postmitotic tissues in higher organisms. Yeast 18: 499–509. 10.1002/yea.701 11284006

[bib79] Maqani N, Fine RD, Shahid M, Li M, Enriquez-Hesles E, Smith JS (2018) Spontaneous mutations in CYC8 and MIG1 suppress the short chronological lifespan of budding yeast lacking SNF1/AMPK. Microb Cell 5: 233–248. 10.15698/mic2018.05.630 29796388 PMC5961917

[bib80] Marino SM, Li Y, Fomenko DE, Agisheva N, Cerny RL, Gladyshev VN (2010) Characterization of surface-exposed reactive cysteine residues in Saccharomyces cerevisiae. Biochemistry 49: 7709–7721. 10.1021/bi100677a 20698499 PMC3061811

[bib81] Matsuo Y, Ikeuchi K, Saeki Y, Iwasaki S, Schmidt C, Udagawa T, Sato F, Tsuchiya H, Becker T, Tanaka K, (2017) Ubiquitination of stalled ribosome triggers ribosome-associated quality control. Nat Commun 8: 159. 10.1038/s41467-017-00188-1 28757607 PMC5534433

[bib82] Matsuo Y, Tesina P, Nakajima S, Mizuno M, Endo A, Buschauer R, Cheng J, Shounai O, Ikeuchi K, Saeki Y, (2020) RQT complex dissociates ribosomes collided on endogenous RQC substrate SDD1. Nat Struct Mol Biol 27: 323–332. 10.1038/s41594-020-0393-9 32203490

[bib83] McDonagh B, Sakellariou GK, Smith NT, Brownridge P, Jackson MJ (2014) Differential cysteine labeling and global label-free proteomics reveals an altered metabolic state in skeletal muscle aging. J Proteome Res 13: 5008–5021. 10.1021/pr5006394 25181601 PMC4227305

[bib84] Meng J, Fu L, Liu K, Tian C, Wu Z, Jung Y, Ferreira RB, Carroll KS, Blackwell TK, Yang J (2021) Global profiling of distinct cysteine redox forms reveals wide-ranging redox regulation in C. elegans. Nat Commun 12: 1415. 10.1038/s41467-021-21686-3 33658510 PMC7930113

[bib85] Menger KE, James AM, Cocheme HM, Harbour ME, Chouchani ET, Ding S, Fearnley IM, Partridge L, Murphy MP (2015) Fasting, but not aging, dramatically alters the redox status of cysteine residues on proteins in Drosophila melanogaster. Cell Rep 11: 1856–1865. 10.1016/j.celrep.2015.05.033 26095360 PMC4508341

[bib86] Mesika R, Reichmann D (2019) When safeguarding goes wrong: Impact of oxidative stress on protein homeostasis in health and neurodegenerative disorders. Adv Protein Chem Struct Biol 114: 221–264. 10.1016/bs.apcsb.2018.11.001 30635082

[bib87] Morgan B, Ezerina D, Amoako TN, Riemer J, Seedorf M, Dick TP (2013) Multiple glutathione disulfide removal pathways mediate cytosolic redox homeostasis. Nat Chem Biol 9: 119–125. 10.1038/nchembio.1142 23242256

[bib88] Naryshkina T, Bruning A, Gadal O, Severinov K (2003) Role of second-largest RNA polymerase I subunit Zn-binding domain in enzyme assembly. Eukaryot Cell 2: 1046–1052. 10.1128/EC.2.5.1046-1052.2003 14555487 PMC219369

[bib89] O’Shea JP, Chou MF, Quader SA, Ryan JK, Church GM, Schwartz D (2013) pLogo: A probabilistic approach to visualizing sequence motifs. Nat Methods 10: 1211–1212. 10.1038/nmeth.2646 24097270

[bib90] Opalinski L, Song J, Priesnitz C, Wenz LS, Oeljeklaus S, Warscheid B, Pfanner N, Becker T (2018) Recruitment of cytosolic J-proteins by TOM receptors promotes mitochondrial protein biogenesis. Cell Rep 25: 2036–2043.e5. 10.1016/j.celrep.2018.10.083 30463002 PMC6280124

[bib91] Pan Y (2011) Mitochondria, reactive oxygen species, and chronological aging: A message from yeast. Exp Gerontol 46: 847–852. 10.1016/j.exger.2011.08.007 21884780

[bib92] Pan Y, Schroeder EA, Ocampo A, Barrientos A, Shadel GS (2011) Regulation of yeast chronological life span by TORC1 via adaptive mitochondrial ROS signaling. Cell Metab 13: 668–678. 10.1016/j.cmet.2011.03.018 21641548 PMC3110654

[bib93] Paulsen CE, Carroll KS (2013) Cysteine-mediated redox signaling: Chemistry, biology, and tools for discovery. Chem Rev 113: 4633–4679. 10.1021/cr300163e 23514336 PMC4303468

[bib94] Pedregosa F, Varoquaux G, Gramfort A, Michel V, Thirion B, Grisel O, Blondel M, Prettenhofer P, Weiss R, Dubourg V, (2011) Scikit-learn: Machine learning in Python. J Mach Learn Res 12: 2825–2830.

[bib95] Perez-Riverol Y, Bai JW, Bandla C, Garcia-Seisdedos D, Hewapathirana S, Kamatchinathan S, Kundu DJ, Prakash A, Frericks-Zipper A, Eisenacher M, (2022) The PRIDE database resources in 2022: A hub for mass spectrometry-based proteomics evidences. Nucleic Acids Res 50: D543–D552. 10.1093/nar/gkab1038 34723319 PMC8728295

[bib96] Pizzino G, Irrera N, Cucinotta M, Pallio G, Mannino F, Arcoraci V, Squadrito F, Altavilla D, Bitto A (2017) Oxidative stress: Harms and benefits for human health. Oxid Med Cell Longev 2017: 8416763. 10.1155/2017/8416763 28819546 PMC5551541

[bib97] Powers RW 3rd, Kaeberlein M, Caldwell SD, Kennedy BK, Fields S (2006) Extension of chronological life span in yeast by decreased TOR pathway signaling. Genes Dev 20: 174–184. 10.1101/gad.1381406 16418483 PMC1356109

[bib98] Qin H, Lu M (2006) Natural variation in replicative and chronological life spans of Saccharomyces cerevisiae. Exp Gerontol 41: 448–456. 10.1016/j.exger.2006.01.007 16516427

[bib99] Rajesh S, Sakamoto T, Iwamoto-Sugai M, Shibata T, Kohno T, Ito Y (1999) Ubiquitin binding interface mapping on yeast ubiquitin hydrolase by NMR chemical shift perturbation. Biochemistry 38: 9242–9253. 10.1021/bi9903953 10413498

[bib100] Reeg S, Grune T (2015) Protein oxidation in aging: Does it play a role in aging progression? Antioxid Redox Signal 23: 239–255. 10.1089/ars.2014.6062 25178482 PMC4507125

[bib101] Rhee SG (2016) Overview on peroxiredoxin. Mol Cells 39: 1–5. 10.14348/molcells.2016.2368 26831451 PMC4749868

[bib102] Rogers AN, Chen D, McColl G, Czerwieniec G, Felkey K, Gibson BW, Hubbard A, Melov S, Lithgow GJ, Kapahi P (2011) Life span extension via eIF4G inhibition is mediated by posttranscriptional remodeling of stress response gene expression in C. elegans. Cell Metab 14: 55–66. 10.1016/j.cmet.2011.05.010 21723504 PMC3220185

[bib103] Rojas M, Farr GW, Fernandez CF, Lauden L, McCormack JC, Wolin SL (2012) Yeast Gis2 and its human ortholog CNBP are novel components of stress-induced RNP granules. PLoS One 7: e52824. 10.1371/journal.pone.0052824 23285195 PMC3528734

[bib104] Rothstein RJ (1983) One-step gene disruption in yeast. Methods Enzymol 101: 202–211. 10.1016/0076-6879(83)01015-0 6310324

[bib105] Roux AE, Leroux A, Alaamery MA, Hoffman CS, Chartrand P, Ferbeyre G, Rokeach LA (2009) Pro-aging effects of glucose signaling through a G protein-coupled glucose receptor in fission yeast. PLoS Genet 5: e1000408. 10.1371/journal.pgen.1000408 19266076 PMC2646135

[bib106] Sakamoto T, Tanaka T, Ito Y, Rajesh S, Iwamoto-Sugai M, Kodera Y, Tsuchida N, Shibata T, Kohno T (1999) An NMR analysis of ubiquitin recognition by yeast ubiquitin hydrolase: Evidence for novel substrate recognition by a cysteine protease. Biochemistry 38: 11634–11642. 10.1021/bi990310y 10512618

[bib107] Saleh A, Collart M, Martens JA, Genereaux J, Allard S, Cote J, Brandl CJ (1998) TOM1p, a yeast hect-domain protein which mediates transcriptional regulation through the ADA/SAGA coactivator complexes. J Mol Biol 282: 933–946. 10.1006/jmbi.1998.2036 9753545

[bib108] Santt O, Pfirrmann T, Braun B, Juretschke J, Kimmig P, Scheel H, Hofmann K, Thumm M, Wolf DH (2008) The yeast GID complex, a novel ubiquitin ligase (E3) involved in the regulation of carbohydrate metabolism. Mol Biol Cell 19: 3323–3333. 10.1091/mbc.e08-03-0328 18508925 PMC2488282

[bib109] Schmitz J, Chowdhury MM, Hanzelmann P, Nimtz M, Lee EY, Schindelin H, Leimkuhler S (2008) The sulfurtransferase activity of Uba4 presents a link between ubiquitin-like protein conjugation and activation of sulfur carrier proteins. Biochemistry 47: 6479–6489. 10.1021/bi800477u 18491921

[bib110] Sevier CS, Qu H, Heldman N, Gross E, Fass D, Kaiser CA (2007) Modulation of cellular disulfide-bond formation and the ER redox environment by feedback regulation of Ero1. Cell 129: 333–344. 10.1016/j.cell.2007.02.039 17448992

[bib111] Shang F, Taylor A (2011) Ubiquitin-proteasome pathway and cellular responses to oxidative stress. Free Radic Biol Med 51: 5–16. 10.1016/j.freeradbiomed.2011.03.031 21530648 PMC3109097

[bib112] Shannon P, Markiel A, Ozier O, Baliga NS, Wang JT, Ramage D, Amin N, Schwikowski B, Ideker T (2003) Cytoscape: A software environment for integrated models of biomolecular interaction networks. Genome Res 13: 2498–2504. 10.1101/gr.1239303 14597658 PMC403769

[bib113] Sievers F, Higgins DG (2021) The clustal omega multiple alignment package. Methods Mol Biol 2231: 3–16. 10.1007/978-1-0716-1036-7_1 33289883

[bib114] Sikorski RS, Hieter P (1989) A system of shuttle vectors and yeast host strains designed for efficient manipulation of DNA in Saccharomyces cerevisiae. Genetics 122: 19–27. 10.1093/genetics/122.1.19 2659436 PMC1203683

[bib115] Simoes V, Cizubu BK, Harley L, Zhou Y, Pajak J, Snyder NA, Bouvette J, Borgnia MJ, Arya G, Bartesaghi A, (2022) Redox-sensitive E2 Rad6 controls cellular response to oxidative stress via K63-linked ubiquitination of ribosomes. Cell Rep 39: 110860. 10.1016/j.celrep.2022.110860 35613580 PMC9215706

[bib116] Snyder NA, Silva GM (2021) Deubiquitinating enzymes (DUBs): Regulation, homeostasis, and oxidative stress response. J Biol Chem 297: 101077. 10.1016/j.jbc.2021.101077 34391779 PMC8424594

[bib117] Spizzo T, Byersdorfer C, Duesterhoeft S, Eide D (1997) The yeast FET5 gene encodes a FET3-related multicopper oxidase implicated in iron transport. Mol Gen Genet 256: 547–556. 10.1007/pl00008615 9413439

[bib118] Stadtman ER (2001) Protein oxidation in aging and age-related diseases. Ann Ny Acad Sci 928: 22–38. 10.1111/j.1749-6632.2001.tb05632.x 11795513

[bib119] Steffen KK, MacKay VL, Kerr EO, Tsuchiya M, Hu D, Fox LA, Dang N, Johnston ED, Oakes JA, Tchao BN, (2008) Yeast life span extension by depletion of 60s ribosomal subunits is mediated by Gcn4. Cell 133: 292–302. 10.1016/j.cell.2008.02.037 18423200 PMC2749658

[bib120] Su Z, Burchfield JG, Yang P, Humphrey SJ, Yang G, Francis D, Yasmin S, Shin SY, Norris DM, Kearney AL, (2019) Global redox proteome and phosphoproteome analysis reveals redox switch in Akt. Nat Commun 10: 5486. 10.1038/s41467-019-13114-4 31792197 PMC6889415

[bib121] Supek F, Bosnjak M, Skunca N, Smuc T (2011) REVIGO summarizes and visualizes long lists of gene ontology terms. PLoS One 6: e21800. 10.1371/journal.pone.0021800 21789182 PMC3138752

[bib122] Tamai KT, Gralla EB, Ellerby LM, Valentine JS, Thiele DJ (1993) Yeast and mammalian metallothioneins functionally substitute for yeast copper-zinc superoxide-dismutase. Proc Natl Acad Sci U S A 90: 8013–8017. 10.1073/pnas.90.17.8013 8367458 PMC47278

[bib123] Tavernarakis N (2008) Ageing and the regulation of protein synthesis: A balancing act? Trends Cell Biol 18: 228–235. 10.1016/j.tcb.2008.02.004 18346894

[bib124] Topf U, Suppanz I, Samluk L, Wrobel L, Boser A, Sakowska P, Knapp B, Pietrzyk MK, Chacinska A, Warscheid B (2018) Quantitative proteomics identifies redox switches for global translation modulation by mitochondrially produced reactive oxygen species. Nat Commun 9: 324. 10.1038/s41467-017-02694-8 29358734 PMC5778013

[bib125] Topf U, Uszczynska-Ratajczak B, Chacinska A (2019) Mitochondrial stress-dependent regulation of cellular protein synthesis. J Cell Sci 132: jcs226258. 10.1242/jcs.226258 31028152

[bib126] Tyanova S, Temu T, Sinitcyn P, Carlson A, Hein MY, Geiger T, Mann M, Cox J (2016) The Perseus computational platform for comprehensive analysis of (prote)omics data. Nat Methods 13: 731–740. 10.1038/nmeth.3901 27348712

[bib127] Ulrich K, Jakob U (2019) The role of thiols in antioxidant systems. Free Radic Biol Med 140: 14–27. 10.1016/j.freeradbiomed.2019.05.035 31201851 PMC7041647

[bib128] Utsugi T, Hirata A, Sekiguchi Y, Sasaki T, Toh-e A, Kikuchi Y (1999) Yeast tom1 mutant exhibits pleiotropic defects in nuclear division, maintenance of nuclear structure and nucleocytoplasmic transport at high temperatures. Gene 234: 285–295. 10.1016/s0378-1119(99)00197-3 10395901

[bib129] van der Reest J, Lilla S, Zheng L, Zanivan S, Gottlieb E (2018) Proteome-wide analysis of cysteine oxidation reveals metabolic sensitivity to redox stress. Nat Commun 9: 1581. 10.1038/s41467-018-04003-3 29679077 PMC5910380

[bib130] Verboven S, Branden KV, Goos P (2007) Sequential imputation for missing values. Comput Biol Chem 31: 320–327. 10.1016/j.compbiolchem.2007.07.001 17920334

[bib131] Vetro JA, Chang YH (2002) Yeast methionine aminopeptidase type 1 is ribosome-associated and requires its N-terminal zinc finger domain for normal function in vivo. J Cell Biochem 85: 678–688. 10.1002/jcb.10161 11968008

[bib132] von der Haar T, Leadsham JE, Sauvadet A, Tarrant D, Adam IS, Saromi K, Laun P, Rinnerthaler M, Breitenbach-Koller H, Breitenbach M, (2017) The control of translational accuracy is a determinant of healthy ageing in yeast. Open Biol 7: 160291. 10.1098/rsob.160291 28100667 PMC5303280

[bib133] Wang YR, Weisenhorn E, MacDiarmid CW, Andreini C, Bucci M, Taggart J, Banci L, Russell J, Coon JJ, Eide DJ (2018) The cellular economy of the Saccharomyces cerevisiae zinc proteome. Metallomics 10: 1755–1776. 10.1039/c8mt00269j 30358795 PMC6291366

[bib134] Weinberger M, Feng L, Paul A, Smith DL, Jr., Hontz RD, Smith JS, Vujcic M, Singh KK, Huberman JA, Burhans WC (2007) DNA replication stress is a determinant of chronological lifespan in budding yeast. PLoS One 2: e748. 10.1371/journal.pone.0000748 17710147 PMC1939877

[bib135] Wilson MA, St Amour CV, Collins JL, Ringe D, Petsko GA (2004) The 1.8-A resolution crystal structure of YDR533Cp from Saccharomyces cerevisiae: A member of the DJ-1/ThiJ/PfpI superfamily. Proc Natl Acad Sci U S A 101: 1531–1536. 10.1073/pnas.0308089100 14745011 PMC341769

[bib136] Winterbourn CC, Hampton MB (2015) Redox biology: Signaling via a peroxiredoxin sensor. Nat Chem Biol 11: 5–6. 10.1038/nchembio.1722 25517384

[bib137] Xiao H, Jedrychowski MP, Schweppe DK, Huttlin EL, Yu Q, Heppner DE, Li J, Long J, Mills EL, Szpyt J, (2020) A quantitative tissue-specific landscape of protein redox regulation during aging. Cell 180: 968–983.e24. 10.1016/j.cell.2020.02.012 32109415 PMC8164166

[bib138] Xie JL, Bohovych I, Wong EOY, Lambert JP, Gingras AC, Khalimonchuk O, Cowen LE, Leach MD (2017) Ydj1 governs fungal morphogenesis and stress response, and facilitates mitochondrial protein import via Mas1 and Mas2. Microb Cell 4: 342–361. 10.15698/mic2017.10.594 29082232 PMC5657825

[bib139] Yang YM, Jung Y, Abegg D, Adibekian A, Carroll KS, Karbstein K (2023) Chaperone-directed ribosome repair after oxidative damage. Mol Cell 83: 1527–1537.e5. 10.1016/j.molcel.2023.03.030 37086725 PMC10164075

[bib140] Yu J, Zhang NN, Yin PD, Cui PX, Zhou CZ (2008) Glutathionylation-triggered conformational changes of glutaredoxin Grx1 from the yeast Saccharomyces cerevisiae. Proteins 72: 1077–1083. 10.1002/prot.22096 18473363

[bib141] Zhang H, Gong W, Wu S, Perrett S (2022) Hsp70 in redox homeostasis. Cells 11: 829. 10.3390/cells11050829 35269451 PMC8909019

[bib142] Zhou Y, Kastritis PL, Dougherty SE, Bouvette J, Hsu AL, Burbaum L, Mosalaganti S, Pfeffer S, Hagen WJH, Forster F, (2020) Structural impact of K63 ubiquitin on yeast translocating ribosomes under oxidative stress. Proc Natl Acad Sci U S A 117: 22157–22166. 10.1073/pnas.2005301117 32855298 PMC7486741

[bib143] Zuo S, Guo Q, Ling C, Chang YH (1995) Evidence that two zinc fingers in the methionine aminopeptidase from Saccharomyces cerevisiae are important for normal growth. Mol Gen Genet 246: 247–253. 10.1007/BF00294688 7862096

